# Early HbA1c Levels as a Predictor of Adverse Obstetric Outcomes: A Systematic Review and Meta-Analysis

**DOI:** 10.3390/jcm13061732

**Published:** 2024-03-17

**Authors:** Laura Mañé, Humberto Navarro, Juan Pedro-Botet, Juan José Chillarón, Silvia Ballesta, Antonio Payà, Verónica Amador, Juana Antonia Flores-Le Roux, David Benaiges

**Affiliations:** 1 Department of Endocrinology and Nutrition, Consorci Hospitalari de Vic, 08500 Vic, Spain; laurams47112@gmail.com; 2 Department of Endocrinology and Nutrition, Consorci Sanitari de l’Alt Penedès i Garraf, 08720 Vilafranca del Pendès, Spainjchillaron@psmar.cat (J.J.C.); silvia.ballesta@gmail.com (S.B.); 3 Department of Endocrinology and Nutrition, Hospital del Mar, 08003 Barcelona, Spain; jpedrobotet@psmar.cat (J.P.-B.); vamador@psmar.cat (V.A.); jaflores@psmar.cat (J.A.F.-L.R.); 4 Department of Medicine, Universitat Autònoma de Barcelona, 08003 Barcelona, Spain; 5 Department of Medicine, Universitat Pompeu Fabra, Campus del Mar, 08003 Barcelona, Spain; apaya@psmar.cat; 6 Cardiovascular Risk and Nutrition Research Group, Hospital del Mar Medical Research Institute (IMIM), 08003 Barcelona, Spain; 7 Department of Gynecology and Obstetrics, Hospital del Mar, 08003 Barcelona, Spain

**Keywords:** glycosylated haemoglobin, hypertension, pregnancy-induced, large for gestational age, macrosomia, pre-eclampsia, pregnancy outcomes

## Abstract

**Background**: The objective was to assess the association between early HbA1c levels and pregnancy complications and whether this relationship is affected when HbA1c thresholds are greater than or less than 39 mmol/mol (5.7%). **Methods**: Electronic searches of the MEDLINE and EMBASE databases up to October 2022 were conducted. We included retrospective and prospective observational studies. The inclusion criteria were as follows: HbA1c measurements taken at <20 weeks’ gestation, singleton pregnancy, and no pre-existing diabetes mellitus. **Results**: We assessed the certainty of the evidence with the GRADE system. We determined the proportion of patients in each group who met the criteria for obstetrical outcomes and pooled data into two subgroups according to the HbA1c threshold: <39 mmol/mol or >39 mmol/mol (5.7%). Sixteen studies with a total of 43,627 women were included. An association between elevated early HbA1c levels and pre-eclampsia, large for gestational age (LGA), macrosomia, and preterm delivery (RR 2.02, 95% CI 1.53–2.66; RR 1.38, 95% CI 1.15–1.66; RR 1.40, 95% CI 1.07–1.83; and RR 1.67, 95% CI 1.39–2.0, respectively) was shown, with a moderate–high grade of certainty. According to the subgroup analysis of all studies, LGA, pre-eclampsia, and labour induction were associated with elevated HbA1c levels only in studies using an HbA1c threshold >39 mmol/mol (5.7%). The association between HbA1c levels and premature birth was statistically significant in studies using both higher and lower HbA1c thresholds. **Conclusions**: Women with high early HbA1c levels below the range of diabetes presented an increased risk of pregnancy complications such as macrosomia, LGA, and pre-eclampsia. An early HbA1c threshold of >39 mmol/mol (5.7%) showed the strongest association with pregnancy complications.

## 1. Introduction

The prevalence of impaired glucose metabolism during pregnancy has increased worldwide in recent years [1,2,3]; the ongoing obesity epidemic, the prevailing sedentary lifestyle, current eating patterns, and advanced maternal age are among the causes. The Hyperglycemia and Adverse Pregnancy Outcomes study, a large international epidemiological study involving 25,000 pregnant women, showed that the risk of maternal and neonatal complications increased linearly with increasing maternal glycaemic levels [4]. The impact of hyperglycaemia during pregnancy, even at levels below the diagnostic range for diabetes, as a continuum of risk for obstetric complications has been proposed by other authors [5,6,7]. However, these studies focused on glycaemic levels in the second half of pregnancy. Assuming that maternal glucose levels behave along a continuum of risk for adverse pregnancy outcomes, the early identification of women with hyperglycaemia by using a simple method is desirable.

HbA1c is formed via the irreversible post-translational nonenzymatic attachment of glucose to haemoglobin and provides information regarding glycaemic control over the previous 2 to 3 months. HbA1c analysis has greater preanalytical stability and fewer day-to-day alterations than glucose analysis and can be easily included in the first antenatal blood tests without fasting, rendering early detection of hyperglycaemia much more feasible with HbA1c tests than with oral glucose tolerance tests [8]. The lack of standardisation in HbA1c measurement limited its use for some time; however, eventually, standardisation in laboratories worldwide led to HbA1c measurement becoming a suitable screening test for diabetes in nonpregnant populations. Less is known about the utility of HbA1c measurement throughout pregnancy. The American Diabetes Association and the International Association of Diabetes and Pregnancy Study Groups (IADPSG) endorse HbA1c measurement as a diagnostic test for unknown pregestational diabetes in early pregnancy [9,10,11]. Currently, an HbA1c level > 6.5% is the recommended diagnostic cut-off for diabetes during pregnancy. However, this is based on data from a nonpregnant population, and the threshold in pregnancy is likely to be lower since HbA1c levels decrease in the first trimester due to changes in erythrocyte lifespan and a decrease in the plasma glucose level [12,13,14]. Indeed, when considering HbA1c measurement as a screening test in early pregnancy to detect significant glucose elevations below the diagnostic range for diabetes, there is little evidence to recommend a specific HbA1c cut-off for intervention. The association between HbA1c levels and adverse pregnancy outcomes among pregnant women without diabetes has been a subject of research over the past decade. A pioneering study conducted in New Zealand [15] showed that an early HbA1c level ≥ 41 mmol/mol (5.9%) was independently associated with an increased risk of obstetric complications. Shortly afterwards, another study in a Spanish population supported the proposed HbA1c threshold as a predictor for adverse pregnancy outcomes, although the association with pregnancy outcomes differed in several ways [16]. In this cohort study, macrosomia was independently related to elevated HbA1c levels, while in the New Zealand study, no correlation of elevated HbA1c levels with birthweight or macrosomia was reported. Moreover, Hughes et al. reported an association between HbA1c levels and preterm delivery, which differed from the findings of Mañe et al. Both studies reported an increased risk of pre-eclampsia in women with an early HbA1c level > 41 mmol/mol (5.9%) and precluded an association between HbA1c levels and caesarean section. Similarly, later authors [17,18] evaluated the role of HbA1c as a predictor of adverse pregnancy outcomes, with considerable heterogeneity among the results. We speculated that these discrepancies could be explained at least in part by a lack of statistical power due to the small sample size of most studies. Accordingly, we acknowledge that conducting a meta-analysis could help clarify these discrepancies, evaluate the real implications of an elevated early HbA1c level below the diagnostic range for diabetes in terms of pregnancy complications, and create opportunities to elucidate the possible benefit of early treatment for women with elevated early HbA1c levels.

Other authors evaluated the role of a broad range of different thresholds, but the findings were inconclusive. An HbA1c cut-off >39 mmol/mol (5.7%) has been widely assessed since it has been classically used to diagnose prediabetes in nonpregnant populations [19,20,21,22,23,24,25], although lower thresholds have been used in other studies [26,27,28,29,30]. Given that maternal glycaemia has been suggested to behave along a continuum of risk for obstetric complications, it would be expected that the association between HbA1c levels and adverse obstetric outcomes would be stronger among studies using higher HbA1c cut-off levels.

Thus, the aim of the present meta-analysis was to ascertain whether an early HbA1c level below the diagnostic range for diabetes is associated with poorer pregnancy outcomes. As a secondary objective, the following question was raised: is the relationship between HbA1c levels and adverse obstetric outcomes affected when HbA1c thresholds are higher or lower than 39 mmol/mol (5.7%)?

## 2. Materials and Methods

The systematic review was reported in accordance with the PRISMA statement [31]. This study was registered in PROSPERO.

### 2.1. Sources and Search Strategy for Identifying Studies

Electronic searches of the MEDLINE and EMBASE databases up to October 2022 were conducted. We designed search algorithms that were adapted to the requirements of each database and that included a combination of controlled vocabulary, search terms, and filters to identify controlled observational studies. The following Medical Subject Headings (MeSH) terms were used: “Glycated Hemoglobin A” AND (“Pregnancy Trimester”, “First OR Pregnancy”) AND (“Fetal Macrosomia” OR “Pre-Eclampsia” OR “Premature Birth” OR “Pregnancy Outcome” OR “Infant, Small for Gestational Age” OR “Gestational Age” OR “Cesarean Section” OR “Labor, Induced” OR “Congenital Abnormalities” OR “Hyperbilirubinemia, Neonatal” OR “Perinatal Death”. We included retrospective and prospective observational studies. No restrictions for geographic location were used. Furthermore, the reference lists of all selected studies were reviewed to identify other studies not captured by the electronic search. The search strategy is shown in Table S1.

### 2.2. Study Selection

The inclusion criteria were women with HbA1c measurements taken before 20 weeks’ gestation, women with singleton pregnancies, and women without pre-existing diabetes mellitus. The following outcomes were evaluated: macrosomia, large for gestational age (LGA), pre-eclampsia, small for gestational age (SGA), preterm delivery, perinatal death, congenital anomaly, caesarean birth, labour induction, hyperbilirubinemia, and foetal respiratory distress. We excluded studies of nonpregnant women, nonhuman studies, studies in which HbA1c measurement was not performed using the National Glycohemoglobin Standardization Program (NGSP), articles for which the full text was not available (only abstract), and posters. When abstracts were classified as uncertain, the full article was reviewed.

### 2.3. Data Extraction, Risk of Bias, and Certainty Assessment

Two independent reviewers (LM and HN) independently selected eligible studies and extracted data using a standardised data extraction form; a third author (DB) was involved to resolve discrepancies. We extracted data from the included studies, including the authors, publication year, study design, cohort size, inclusion criteria, early HbA1c threshold, and occurrence of adverse outcomes. A weighted kappa was used to determine agreement between reviewers and the reliability of the article selection [32].

We assessed the risk of bias using the ROBINS-I tool, which was designed to evaluate the selection of study groups and the comparability of groups and to ascertain either the exposure or outcome [33]. The possibility of publication bias was estimated by the visual inspection of the funnel plot and using Begg’s test and Egger’s test.

Two authors (LM and HN) independently estimated the certainty of the evidence. We used the five GRADE considerations (study limitations, consistency of effect, imprecision, indirectness, and publication bias) to determine the certainty of the body of evidence as it related to the studies from which data for the prespecified outcomes were extracted for the meta-analyses [34]. We classified the certainty of evidence as high, moderate, low, or very low. We applied the methods and recommendations described in Sections 8.5 and 8.7 and Chapters 11 and 12 of the *Cochrane Handbook for Systematic Reviews of Interventions* [33]. We used GRADEpro GDT Copyright © 2024 software to prepare the ‘Summary of findings’ tables (GRADEpro GDT 2015) [34,35].

### 2.4. Data Synthesis

We determined the proportion of patients in each group who met the criteria for obstetrical outcomes. We pooled the data into two subgroups according to the HbA1c cut-off level: studies using an HbA1c threshold <39 mmol/mol (5.7%) and those using an HbA1c threshold >39 mmol/mol (5.7%). We calculated relative risks (RRs) with 95% CIs for the different pregnancy outcomes. We used the χ² statistic to assess heterogeneity between studies and the I2 statistic to assess the extent of inconsistency. If there was significant (*p* < 0.05) heterogeneity, a random-effects statistical model was used to confirm the case results. A fixed-effects model for the calculations of summary estimates and their 95% CIs was also applied unless there was significant heterogeneity. For all tests, p < 0.05 was considered to indicate statistical significance.

The meta-analysis was performed with RevMan 5.3 software.

## 3. Results

### 3.1. Study Selection

**Figure 1:**
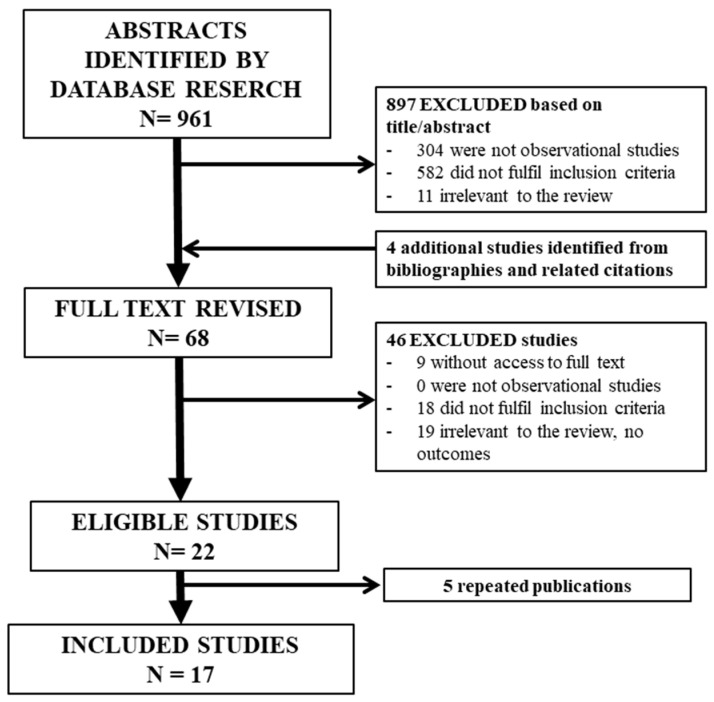
Study screening and data extraction process. We identified studies through electronic searches, and 4 additional studies were identified after reviewing the citations of relevant studies. After excluding ineligible and repeated studies, 16 studies met the inclusion criteria.

We describe the eligibility process in a PRISMA flow diagram (Figure 1), and the complete checklist is included in the Supplementary Materials. We identified studies through electronic searches, and four additional studies were identified by reviewing the citations of relevant studies. Two studies [16,36] reported overlapping data, as they followed the same cohort from April 2013 to September 2015 and from April 2013 to October 2016. To assess the associations of HbA1c levels with macrosomia, LGA, and pre-eclampsia, we used data from a later study [36], as it included a larger cohort, and the first study was excluded. To assess the association of HbA1c levels with the other obstetric outcomes, we obtained data from a prior study [16] since these outcomes were not analysed in the second study. Ultimately, 16 studies met the inclusion criteria. The two reviewers achieved good agreement in the selection of studies (weighted kappa = 0.962, 95% CI = 0.889 to 1.000).

### 3.2. Study and Patient Characteristics

The main characteristics of the 16 included studies, which included a total of 43,627 singleton pregnant women, are detailed in Table 1. Most studies reported HbA1c levels at <20 weeks’ gestation, although two studies reported HbA1c levels at <14 weeks’ gestation [26,30], one reported HbA1c levels at <13 weeks’ and 6 days’ gestation [23,25], and two other articles [20,29] did not specify the week of gestation but reported that HbA1c measurement was conducted in the first trimester. There were three studies from Europe [16,21,29], five from the United States of America [19,22,23,24,25], four from Australia and New Zealand [15,17,18,27], and the remaining four studies focused on other countries [20,26,28,30], mostly in Asia. Women who received treatment for GDM were excluded from some studies [15,24,30]. Conversely, one study [17] exclusively included women diagnosed with GDM. In addition, the study by Immanuel et al. [21] included only women with a BMI > 29 kg/m^2^, and Dillon et al. [23] included only women with a BMI > 40 kg/m^2^. On the other hand, the study by Amylidi et al. included only high-risk women with at least one risk factor for pre-existing diabetes [29].

Seven studies [19,20,21,22,23,24,25] evaluated the impact of an HbA1c cut-off >39 mmol/mol (5.7%), four studies [15,16,17,18] used a cut-off >41 mmol/mol (5.9%), and five studies reported using other thresholds, including 33 mmol/mol (5.2%) [26,28], 37 mmol/mol (5.5%) [30], 38 mmol/mol (5.6%) [27], and 42 mmol/mol (6%) [29].

### 3.3. Quality of the Included Studies

We assessed the risk of bias using the ROBINS-I tool [33]. Six studies showed a low risk of bias, and eleven studies were rated as having a moderate risk of bias (Table S2). Major sources of concern included the selection of participants since some authors only selected women with a high baseline risk of obstetric complications [17,20,23,29] and missing data due to the retrospective nature of some studies [17,18,22,23,29]. Moreover, as none of the included studies were randomised control trials, the “Classification of interventions” and “Deviations from the intended intervention” domains could not be assessed.

### 3.4. Publication Bias

Funnel plots are shown in the Supplementary Materials (Figures S1–S12). No publication bias was detected, except for preterm delivery (Egger’s test *p* value = 0.041).

### 3.5. Synthesis of Results

Figures S1–S12 show the evaluated forest plots for each outcome. The heterogeneity test detected statistically significant variability among the included studies, and a random-effects model was used to pool data for the following outcomes: macrosomia, LGA, SGA, caesarean section, labour induction, and foetal respiratory distress. For the other outcomes, a fixed-effects model was employed, as no significant variability among the studies was identified. Moreover, a summary of the findings for each outcome after the pooled analysis of the studies and the certainty of the body of evidence using the GRADE system are shown in Table 2.

An association between elevated early HbA1c levels and the development of pre-eclampsia was shown, with a high degree of certainty (RR 2.02, 95% CI 1.53 to 2.66).

A high early HbA1c level was also related to the occurrence of LGA, macrosomia, and preterm delivery (RR 1.38, 95% CI 1.15 to 1.66; RR 1.40, 95% CI 1.07–1.83; and RR 1.67, 95% CI 1.39 to 2.0, respectively), with a moderate grade of evidence.

Major congenital anomalies and perinatal death were associated with HbA1c levels above the threshold (RR 2.38, 95% CI 1.46 to 3.87 and RR 2.34, 95% CI 1.33 to 4.12, respectively) but with a very low grade of evidence.

There was no association between elevated HbA1c levels and labour induction (RR 1.26, 95% CI 0.98 to 1.62), with a moderate quality of evidence.

There were also no significant differences in the rates of SGA, caesarean section delivery, hyperbilirubinemia, or foetal respiratory distress between women with high early HbA1c levels and those with low HbA1c levels (RR 1.03 95% CI 0.72 to 1.47), RR 1.12, 95% CI 0.99 to 1.27, RR 1.24, 95% CI 0.99 to 1.57, and RR 1.74, 95% CI 0.23 to 13.27), with a low or very low grade of evidence.

### 3.6. Subgroup Analysis for Study Design

A subgroup analysis was carried out to ascertain the effect of the early HbA1c cut-off level (Figures S1–S11). Regarding LGA, pre-eclampsia and labour induction, the association between HbA1c levels and these pregnancy complications was significant only among studies using an HbA1c threshold >39 mmol/mol (5.7%). Conversely, the occurrence of macrosomia was not significantly related to whether an HbA1c threshold > or <39 mmol/mol (5.7%) was used in the studies. The association between HbA1c levels and premature birth was significant among studies using an HbA1c threshold >39 mmol/mol (5.7%) and those using a cut-off <39 mmol/mol (5.7%). There was no association between HbA1c levels and SGA or caesarean section, regardless of the HbA1c cut-off.

## 4. Discussion

The present meta-analysis included 16 studies (9 retrospective and 7 prospective studies) covering a total of 43,627 singleton pregnant women and provided evidence on the role of early HbA1c levels as a predictor of pregnancy outcomes.

Our first objective was to assess whether early HbA1c levels could be used as a predictor of poor pregnancy outcomes. An association was shown between early HbA1c levels outside the diagnostic range for diabetes and LGA (OR 1.38, 95% CI 1.15–1.66), macrosomia (OR 1.40, 95% CI 1.07–1.83), pre-eclampsia (OR 2.02, 95% CI 1.53–2.66), preterm birth (OR 1.67, 95% CI 1.39–2.00), major congenital anomalies (OR 2.38, 95% CI 1.46–3.87), and perinatal death (OR 2.34, 95% CI 1.33–4.12).

Previous studies have highlighted an association between hyperglycaemia (both in women with GDM and pregestational diabetes) and macrosomia, LGA, pre-eclampsia, and preterm delivery [4,37,38]. Moreover, the results of the HAPO [4] study also revealed a clear association between second-trimester HbA1c levels below the diagnostic range for diabetes and adverse obstetric outcomes, although the association with glucose concentrations was significantly stronger than that with HbA1c levels.

Our findings extend this relationship to early HbA1c levels. It should be emphasised that the high degree of certainty (GRADE scale score: 3–4 = moderate–high certainty) achieved and the low–moderate risk of bias determined by the ROBINS-I tool for the association of HbA1c levels with macrosomia, LGA, pre-eclampsia, and preterm delivery support the robustness of the present results. On the other hand, the observed associations between HbA1c levels and perinatal death and congenital anomalies should be interpreted with caution, as the degree of certainty determined by the GRADE scale was very low. In this respect, previous studies have also suggested an association between mildly abnormal glucose metabolism during pregnancy and an increased risk for some congenital abnormalities [39]. The risk of perinatal death is an outcome that requires cautious interpretation given its low frequency. Although perinatal death has been shown to be associated with pre-existing diabetes, its relationship with a lesser degree of hyperglycaemia remains unclear [5,40].

Overall, no associations were found between HbA1c levels and SGA, caesarean section, labour induction, hyperbilirubinemia, or foetal respiratory distress. It should be noted that the degree of certainty for these conditions was low or very low (GRADE scale: 1–2 points). Likewise, most studies that have evaluated the relationship between GDM and SGA have also failed to find an association [41]. A plausible explanation is the existence of other risk factors, such as low socioeconomic status, nulliparity, access to health care, smoking status, caffeine intake, maternal underweight status, hypertensive disorders, and comorbidities such as HIV infection, that may play a more relevant role in the occurrence of SGA than the degree of maternal dysglycaemia at baseline [42,43]. The relationship between foetal respiratory distress and maternal blood glucose levels remains unclear. Previous studies have shown similar frequencies of foetal respiratory distress and neonatal jaundice among GDM groups in comparison to control groups and of foetal respiratory distress in women with mild GDM in intervention groups versus standard prenatal care groups [44]. Overall, as suggested by van der Tuuk et al., these conditions (caesarean section delivery, labour induction, hyperbilirubinemia, and foetal respiratory distress) seem to depend on a wide range of factors apart from glycaemic control [45,46,47].

Another question addressed by the present meta-analyses is as follows: which first-trimester HbA1c threshold should be used for identifying women at high risk of adverse obstetric outcomes? As mentioned before, after the studies of Hughes et al. and Mañé et al. [15,16] were conducted, a number of studies evaluating a wide range of first-trimester HbA1c thresholds in pregnant women emerged, yet conflicting results were obtained. The HAPO study demonstrated that higher second-trimester maternal HbA1c levels outside the diagnostic range for diabetes were associated with a greater frequency of adverse perinatal outcomes such as LGA, caesarean section, and cord serum C-peptide levels in the 90th percentile [4]. We hypothesised that the same relationship would be observed with early HbA1c levels. Hence, a secondary objective of this work was to establish whether an HbA1c threshold >39 mmol/mol (5.7%) is associated with a greater risk of adverse obstetric outcomes than a lower HbA1c threshold. We selected a threshold of 39 mmol/mol (5.7%) since this cut-off level is classically used to diagnose prediabetes in the general population.

Again, the results of this meta-analysis are in line with our initial hypothesis. In the subgroup analysis, LGA (OR 1.76, 95% CI 1.49–2.08), pre-eclampsia (OR 2.45, 95% CI 1.75–3.43), and labour induction (OR 1.61, 95% CI 1.28–2.02) were associated with elevated HbA1c levels only among studies using an HbA1c threshold >39 mmol/mol (5.7%). These findings are in line with those of the HAPO study [4] and indicate that hyperglycaemia behaves as a continuous variable where higher levels are associated with a greater risk of obstetric complications. Furthermore, gestational diabetes mellitus has been proven to be an independent risk factor for pre-eclampsia, after adjusting for confounders [48,49]. The metabolomic studies undertaken in the serum of women at 11–13 weeks of gestation who later developed late-onset pre-eclampsia identified that insulin resistance and metabolic syndrome, mitochondrial dysfunction, disturbance of energy metabolism, oxidative stress, and lipid dysfunction are present in late-onset pre-eclampsia [50], suggesting that disturbances can be identified early in the disease process. Carlsen et al. showed that, among women with no recorded diabetes, higher HbA1c levels at 18 gestational weeks were associated with an increased risk of pre-eclampsia. Pre-eclampsia increased by 20% per unit increase of HbA1c (95% CI: 5%, 37%) [51] and, within the highest quartile of HbA1c (35mmol/mol or greater), increasing HbA1c was related to shorter pregnancy durations and an increased risk of pre-eclampsia and preterm delivery. Conversely, the occurrence of macrosomia was not significantly related to whether studies used an HbA1c threshold > or <39 mmol/mol (5.7%) (OR 1.20, 95% CI 0.98–1.47 and OR 2.06, 95% CI 0.93–4.55, respectively). However, it should be noted that although the association between HbA1c levels and macrosomia was not significant among the studies with elevated HbA1c thresholds, the RR was high (2.06), and there was remarkable heterogeneity (*p* = 0.0004, I2 87%).

The association between HbA1c levels and preterm birth was significant among both studies using an HbA1c threshold >39 mmol/mol (5.7%) (OR 1.71, 95% CI 1.32–2.21) and those using a cut-off <39 mmol/mol (5.7%) (OR 1.63, 95% CI 1.27–2.10). The fact that most of the studies did not specify whether preterm births were spontaneous or iatrogenic makes it difficult to speculate on the reasons for this association. It could be proposed that hyperglycaemia below the diagnostic range for diabetes has the potential to modify biological processes that contribute to the pathogenesis of preterm delivery, such as altered vascularity or cervicovaginal microbiota. An elevated HbA1c level could also be suggestive of a metabolic syndrome phenotype, and the inflammation and prothrombotic state associated with this clinical scenario could predispose women to preterm birth.

To the best of our knowledge, this is the first meta-analysis addressing the role of early HbA1c levels as a predictor of obstetric outcomes. An extensive systematic literature search was conducted, including 16 studies (43,627 women) without time, country, or ethnicity restrictions, and several outcomes were evaluated. Two independent reviewers extracted the data and achieved good agreement in the selection of studies. The risk of bias was assessed using the ROBINS-I tool, and most studies presented a moderate risk and few applicability concerns. Additionally, we carried out a subgroup analysis to assess whether the association between HbA1c levels and adverse obstetric outcomes was affected when HbA1c thresholds were higher or lower than 39 mmol/mol (5.7%).

### Limitations

This meta-analysis was not without limitations. The significant heterogeneity in terms of HbA1c thresholds and pregnancy outcomes must be considered. Additionally, some of the included studies were retrospective. The small sample size of some of the studies together with the fact that women were recruited worldwide may influence the results since ethnic differences in the association between first-trimester HbA1c levels and the occurrence of obstetric complications and in the prevalence of dysglycaemia have been reported [36]. Ethnic differences in the prevalence of dysglycaemia may be due to genetic factors affecting insulin resistance, diet, lifestyle, sociocultural factors, health care access/utilisation, or even provider discrimination. Although we only included studies that employed HbA1c levels determined by standardised methods, information on the performance of each laboratory was not available, and some studies were unable to take the presence of variants of haemoglobin or iron deficiency into account, which could impact HbA1c levels. The exclusion of letters to the editors, posters, and conference abstracts may have caused publication bias, and due to the linguistic skills of the two reviewers, we included only English and Spanish articles, which might have led to language bias. No contact was established with the authors if an article was published in a language other than Spanish or English. The fact that some studies excluded women who received treatment for GDM [15,24,30] and one study [17] exclusively included women diagnosed with GDM could also have affected the results, as an intervention in this group of patients could modify pregnancy outcomes. A GDM diagnosis is associated with adverse pregnancy outcomes, and GDM treatment has been repeatedly shown to minimise the risk of obstetric complications [44,45,46,47,48,52]. Additionally, two studies included only women with BMIs > 29 kg/m^2^ and >40 kg/m^2^, and a high pregestational BMI is a known risk factor for macrosomia, LGA, pre-eclampsia, caesarean section delivery, and other obstetric complications [53,54]. The discrepancies described in this meta-analysis could also be explained by disparities between the included studies in terms of the ethnic origin of the populations; variations in the gestational period in which HbA1c testing was performed; and disparities between countries in terms of both criteria for GDM diagnosis and treatment, and for the diagnosis and management of milder states of hyperglycaemia or prediabetes.

## 5. Conclusions

Overall, this meta-analysis provided further evidence of the potential role of early HbA1c levels as a marker of pregnancy complications. Special emphasis was placed on a subgroup of women with early HbA1c levels below the diagnostic range for diabetes; these women had an almost 50% higher risk of macrosomia and preterm delivery, and an almost twofold increased risk of pre-eclampsia and LGA and could have been identified in the first trimester, thereby providing an opportunity for early intervention. Furthermore, an early HbA1c threshold >39 mmol/mol (5.7%) showed the strongest association with adverse pregnancy outcomes. Randomised trials are warranted to evaluate whether early intervention in women with HbA1c levels above the threshold translates to improved obstetric outcomes; in this instance, the paradigm of the management of hyperglycaemia during pregnancy would change.

## Figures and Tables

**Table 1:** Summary of the included studies.

Author, Year	Included studies (N = 43,627)	Country	Study Design	Eligibility Criteria	HbA1c Cut-off Point
Hugues RC, 2014 [15]	8497	New Zealand	Prospective	HbA1c measurement at ≤20 weeks’ gestation, no pre-existing diabetes, HbA1c levels < 47.5 mmol/mol (6.5%). Women who received treatment for gestational diabetes (GDM) or were lost to follow-up were excluded.	41 mmol/mol ≥5.9%
Fong A, 2014 [22]	526	USA	Retrospective	HbA1c measurement at ≤20 weeks’ gestation, no pre-existing diabetes, HbA1c level < 47.5 mmol/mol (6.5%).	39 mmol/mol ≥5.7%
Hammouda S, 2015 [20]	1106	Saudi Arabia	Prospective	HbA1c measurement during the first trimester. Women with systemic diseases known to cause birth defects or pre-existing diabetes were excluded. Women with 1T-HbA1c levels > 47.5 mmol/mol (6.5%) were not excluded.	39 mmol/mol ≥5.7%
Amylidi S, 2016 [29]	208	Switzerland	Retrospective	Women with at least one risk factor for pre-existing diabetes who had an HbA1c measurement in the first trimester. No pre-existing diabetes, HbA1c level < 47.5 mmol/mol (6.5%).	42 mmol/mol ≥6.0%
Osmundson SS, 2016 [25]	2812	USA	Retrospective	Delivered at more than 20 weeks’ gestation. HbA1c measurement at ≤13^6/7^ weeks’ gestation. No pre-existing diabetes, HbA1c level < 47.5 mmol/mol (6.5%), and completed GDM testing.	39 mmol/mol ≥5.7%
Sweeting A, 2017 [17]	3098	Australia	Retrospective	HbA1c measurement at the time of GDM diagnosis. No pre-existing diabetes, HbA1c level < 47.5 mmol/mol (6.5%), FPG level <126 mg/L.	41 mmol/mol ≥5.9%
Mane L, 2017 [16]	1228	Spain	Prospective	HbA1c measurement at ≤20 weeks’ gestation; no pre-existing diabetes, HbA1c level < 47.5 mmol/mol (6.5%), FPG level < 126 mg/L. No miscarriage or voluntary pregnancy termination and not lost to follow-up.	41 mmol/mol ≥5.9%
Poo ZX, 2018 [26]	151	Singapore	Prospective	HbA1c measurement at ≤14 weeks’ gestation; no pre-existing diabetes, HbA1c level <47.5 mmol/mol (6.5%). No termination of pregnancy and no haemoglobinopathies. Completed GDM testing.	33 mmol/mol ≥5.2%
Yu H, 2019 [28]	1836	China	Prospective	HbA1c measurement at 12–20 weeks’ gestation. No pre-existing diabetes, HbA1c level < 47.5 mmol/mol (6.5%), FPG level < 126 mg/L or 2 h glucose levels < 200 mg/L. No chronic hypertension, kidney disease or other significant chronic medical diseases, or history of multiple abortions, smoking, and excessive alcohol consumption.	33 mmol/mol ≥5.2%
Chen L, 2019 [19]	7020	USA	Retrospective	Women with continuous enrolment in KPWA from 12 weeks’ gestation through 28 days after delivery who underwent GDM testing. HbA1c measurement at ≤20 weeks’ gestation, no pre-existing diabetes, HbA1c level < 47.5 mmol/mol (6.5%).	39 mmol/mol ≥5.7%
Mañé L, 2019 [36]	1882	Spain	Prospective	HbA1c measurement at ≤20 weeks’ gestation, no pre-existing diabetes, HbA1c level < 47.5 mmol/mol (6.5%), FPG level < 126 mg/L, not lost to follow-up. Women belonging to other ethnicities (not Caucasian, Latin American, South Central Asian, Moroccan, or East Asian) and those for whom information about ethnicity was missing were not included	31 mmol/mol (≥5.0%) to 42 mmol/mol (≥6.0%)
Immanuel JJ, 2020 [21]	869	Multicentric (9 European countries)	A post hoc analysis of vitamin D levels and lifestyle interventions for GDM prevention (DALI trial)	HbA1c measurement at ≤20 weeks’ gestation, no pre-existing diabetes, HbA1c levels < 47.5 mmol/mol (6.5%), FPG level < 126 mg/L, BMI ≥ 29 kg/m^2^. Women with psychiatric and chronic medical conditions, language barriers, or the inability to perform lifestyle interventions were ineligible.	39 mmol/mol ≥5.7%
Lim Y, 2021 [18]	10,869	New Zealand	Retrospective	HbA1c measurement at ≤20 weeks’ gestation, no pre-existing diabetes, HbA1c levels < 47.5 mmol/mol (6.5%).	41 mmol/mol ≥5.9%
Jamieson EL, 2021 [27]	396	Australia	Prospective	≤20 weeks’ gestation, no pre-existing diabetes, completed GDM testing.	38 mmol/mol ≥5.6%
Punnose J, 2022 [30]	1618	India	Retrospective	HbA1c measurement at ≤14 weeks’ gestation, completed GDM testing and delivered in our hospital. Women with pre-existing diabetes mellitus or a first-trimester HbA1c level ≥ 47.5 mmol/mol (6.5%), GDM diagnosis after 24 gestational weeks or β thalassemia trait were excluded.	37 mmol/mol ≥5.5%
Dillon J, 2022 [23]	118	USA	Retrospective	Entry at prenatal care ≤ 13 weeks’ and 6 days’ gestation and delivery at term (≥ 7 weeks). HbA1c measurement at <20 weeks’ gestation, maternal BMI ≥4 0 kg/m^2,^ and no foetal anomalies. Women with pre-existing diabetes mellitus or a 1T-HbA1c level ≥ 47.5 mmol/mol (6.5%) were excluded.	39 mmol/mol ≥5.7%
Bender WR, 2022 [24]	2621	USA	Retrospective	HbA1c measurement at <17 weeks’ gestation completed GDM testing and delivered at our institution. Women with pre-existing diabetes mellitus, an HbA1c level ≥ 47.5 mmol/mol (6.5%) or a GDM diagnosis were excluded.	39 mmol/mol ≥5.7%

GDM: gestational diabetes mellitus. FPG: fasting plasma glucose. BMI: body mass index. LGA: large for gestational age. RDS: respiratory distress syndrome.

**Table 2:** Summary of findings.

Outcomes	Total Events		Relative Effect (95% CI)	Number of Participants (Number of Studies)	Certainty of Evidence (GRADE System)				
	HbA1c Lower	HbA1c Higher			Risk of Bias	Inconsistency	Indirectness	Imprecision	Certainty
LGA	3823/35,129 (10.9%)	220/1546 (14.2%)	1.38 (95% CI 1.15 to 1.66)	36,675 (12 [15,17,18,19,21,23,24,25,26,27,30,36])	Serious ^a^	Serious ^b^	Not serious	Not serious	⨁⨁⨁◯ Moderate
Macrosomia	2043/28,779 (7.1%)	134/1379 (9.7%)	1.40 (95% CI 1.07 to 1.83)	30,158 (10 [15,17,19,21,22,23,24,28,30,36])	Serious ^c^	Serious ^d^	Not serious	Not serious	⨁⨁⨁◯ Moderate
Pre-eclampsia	912/27,448 (3.3%)	50/755 (6.6%)	2.02 (95% CI 1.53 to 2.66)	28,203 (5 [15,18,19,21,36])	Serious ^e^	Not serious	Not serious	Not serious	⨁⨁⨁⨁ High
SGA	3579/32,294 (11.1%)	180/1336 (13.5%)	1.03 (95% CI 0.72 to 1.47)	33,630 (10 [15,16,17,18,19,21,22,24,26,30])	Serious ^f^	Serious ^g^	Not serious	Not serious	⨁⨁◯◯ Low
Preterm delivery	1801/31,802 (5.7%)	116/1280 (9.1%)	1.67 (95% CI 1.39 to 2.00)	33,082 (9 [15,16,17,18,19,21,24,26,30])	Serious ^h^	Not serious	Not serious	Not serious	⨁⨁⨁◯ Moderate
Caesarean section	6606/24,566 (26.9%)	435/1388 (31.3%)	1.12 (95% CI 0.99 to 1.27)	25,954 (12 [15,16,17,19,21,22,23,24,25,26,29,30])	Serious ^i^	Serious ^j^	Not serious	Not serious	⨁⨁◯◯ Low
Labour induction	6486/28,566 (22.7%)	278/901 (30.9%)	1.26 (95% CI 0.98 to 1.62)	29,467 (5 [15,18,19,21,25])	Serious ^k^	Serious ^l^	Not serious	Not serious	⨁⨁⨁◯ Moderate
Major congenital anomalies	146/10,004 (1.5%)	43/1100 (3.9%)	2.38 (95% CI 1.46 to 3.87)	11,104 (3 [15,20,28])	Serious ^m^	Not serious	Serious ^n^	Serious ^o^	⨁◯◯◯ Very low
Perinatal death	161/19,810 (0.8%)	14/1082 (1.3%)	2.34 (95% CI 1.33 to 4.12)	20,892 (3 [15,18,28])	Serious ^p^	Not serious	Serious ^q^	Serious ^o^	⨁◯◯◯ Very low
Hyperbilirubinemia	353/5585 (6.3%)	93/1189 (7.8%)	1.24 (95% CI 0.99 to 1.57)	6774 (5 [21,22,24,28,30])	Serious ^r^	Not serious	Not serious	Serious ^s^	⨁⨁◯◯ Low
Foetal respiratory distress	160/2686 (6.0%)	20/768 (2.6%)	1.74 (95% CI 0.23 to 13.27)	3454 (2 [28,30])	Not serious ^t^	Serious ^u^	Serious ^v^	Serious ^w^	⨁◯◯◯ Very low

LGA: large for gestational age. SGA: small for gestational age. ^a^ More than half of the included studies (7/12) had a moderate risk of bias. ^b^ A heterogeneity test was performed to detect variability among the included studies (*p* < 0.04, I2 47%). ^c^ More than half of the included studies (6/10) had a moderate risk of bias. ^d^ A heterogeneity test was performed to detect variability among the included studies (*p* < 0.03, I2 52%). ^e^ More than half of the included studies (3/5) had a moderate risk of bias. ^f^ More than half of the included studies (6/10) had a moderate risk of bias. ^g^ A heterogeneity test was performed to detect variability among the included studies (*p* < 0.0003, I2 71%). ^h^ More than half of the included studies (5/9) had a moderate risk of bias. ^i^ More than half of the included studies (7/12) had a moderate risk of bias. ^j^ A heterogeneity test was performed to detect variability among the included studies (*p* < 0.03, I2 50%). ^k^ More than half of the included studies (3/5) had a moderate risk of bias. ^l^ A heterogeneity test was performed to detect variability among the included studies (*p* < 0.0001, I2 84%). ^m^ More than half of the included studies (2/3) had a moderate risk of bias. ^n^ Yu et al. did not define “major congenital anomalies”. ^o^ The n of events was small. ^p^ More than half of the included studies (2/3) had a moderate risk of bias. ^q^ Yu et al. did not define “perinatal death”. ^r^ More than half of the included studies (3/5) had a moderate risk of bias. ^s^ The n of patients and events was small. ^t^ No more than half of the included studies (1/2) had a moderate risk of bias. ^u^ A heterogeneity test was performed to detect variability among the included studies (*p* < 0.002, I2 89%). ^v^ Yu et al. did not define “foetal respiratory distress”. ^w^ The n of patients and events was small and the confidence intervals were wide (95% CI 0.23 to 13.27).

## Data Availability

This study was registered in PROSPERO, number CRD42022338125. The full dataset is available online (https://www.crd.york.ac.uk/prospero/display_record.php?ID=CRD42022338125, accessed on 20 December 2022). The original contributions presented in the study are included in the article/Supplementary Materials, further inquiries can be directed to the corresponding author/s. DB is the guarantor of this work and, as such, has full access to all the data in the study and takes responsibility for the integrity of the data and the accuracy of the data analysis.

## Supplementary Materials

The following supporting information can be downloaded at https://www.mdpi.com/article/10.3390/jcm13061732/s1. Table S1: Search strategy; Table S2: Risk of bias for the included observational studies determined using the ROBINS-I tool; and Figures S1–S12: Forest plot showing the results of meta-analyses for each outcome and funnel plots and Begg’s and Egger’s tests assessing publication bias.
