# Supplementary material for: Early HbA1c Levels as a Predictor of Adverse Obstetric Outcomes: A Systematic Review and Meta-Analysis
Source: J Clin Med. 2024 Mar 17;13(6):1732. doi: 10.3390/jcm13061732 (PMC10970986; doi:10.3390/jcm13061732)
Supplement: Supplementary file 1 [file jcm-13-01732-s001.zip › Figures S1-12.pptx]

## Slide 1
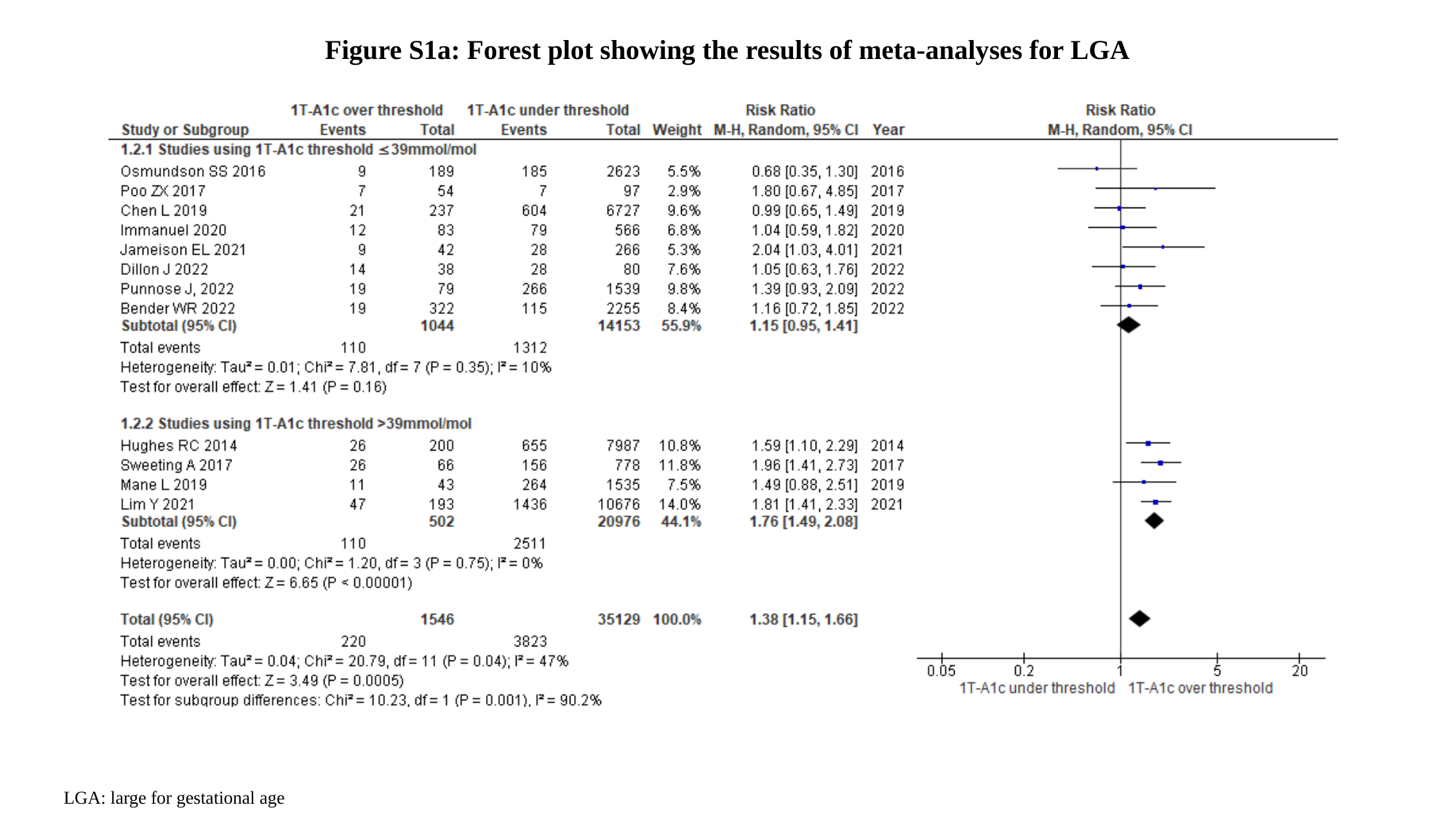

# Figure S1a: Forest plot showing the results of meta-analyses for LGA
LGA: large for gestational age

## Slide 2
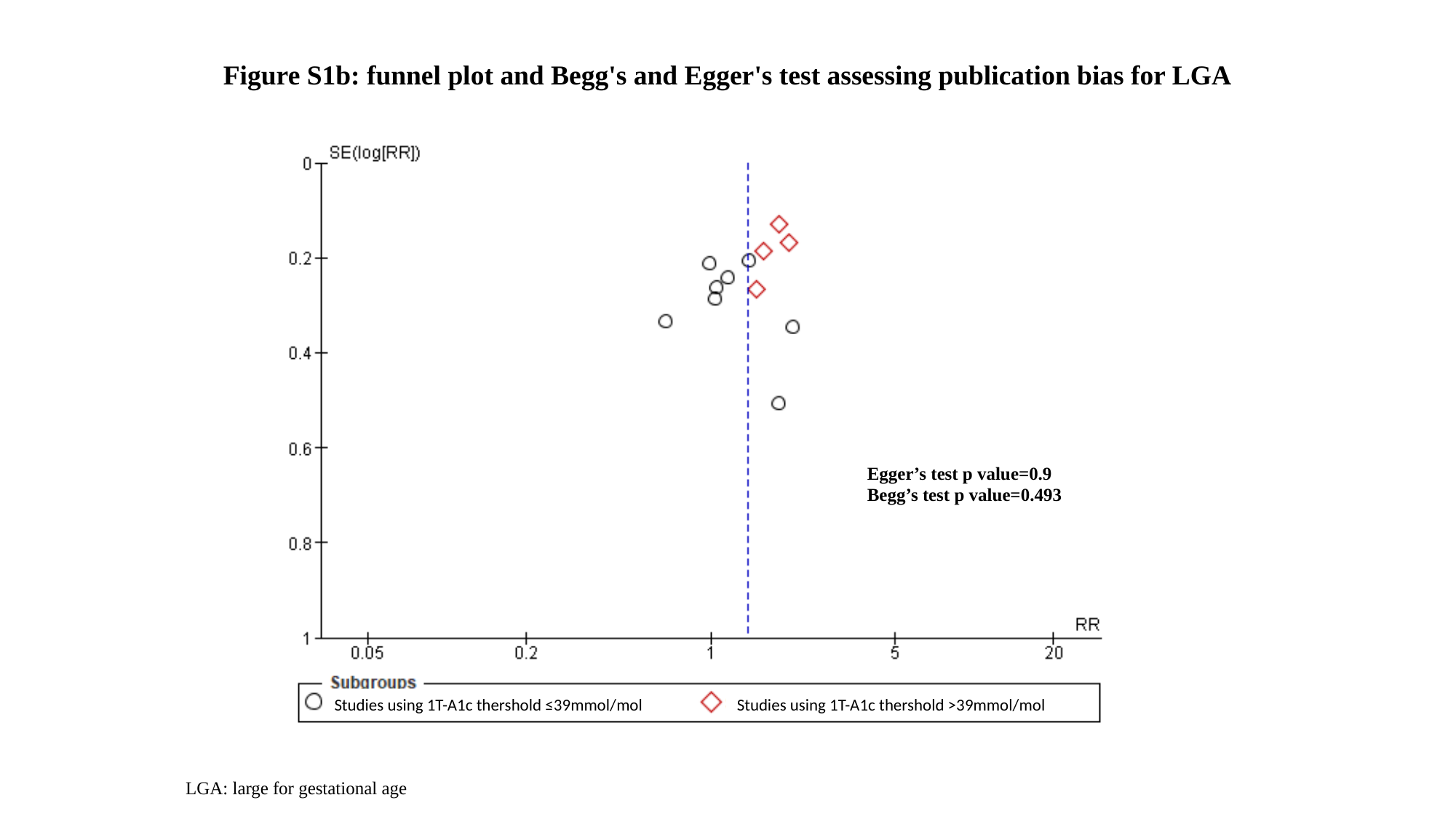

# Figure S1b: funnel plot and Begg's and Egger's test assessing publication bias for LGA
Egger’s test p value=0.9
Begg’s test p value=0.493
Studies using 1T-A1c thershold >39mmol/mol
Studies using 1T-A1c thershold ≤39mmol/mol
LGA: large for gestational age

## Slide 3
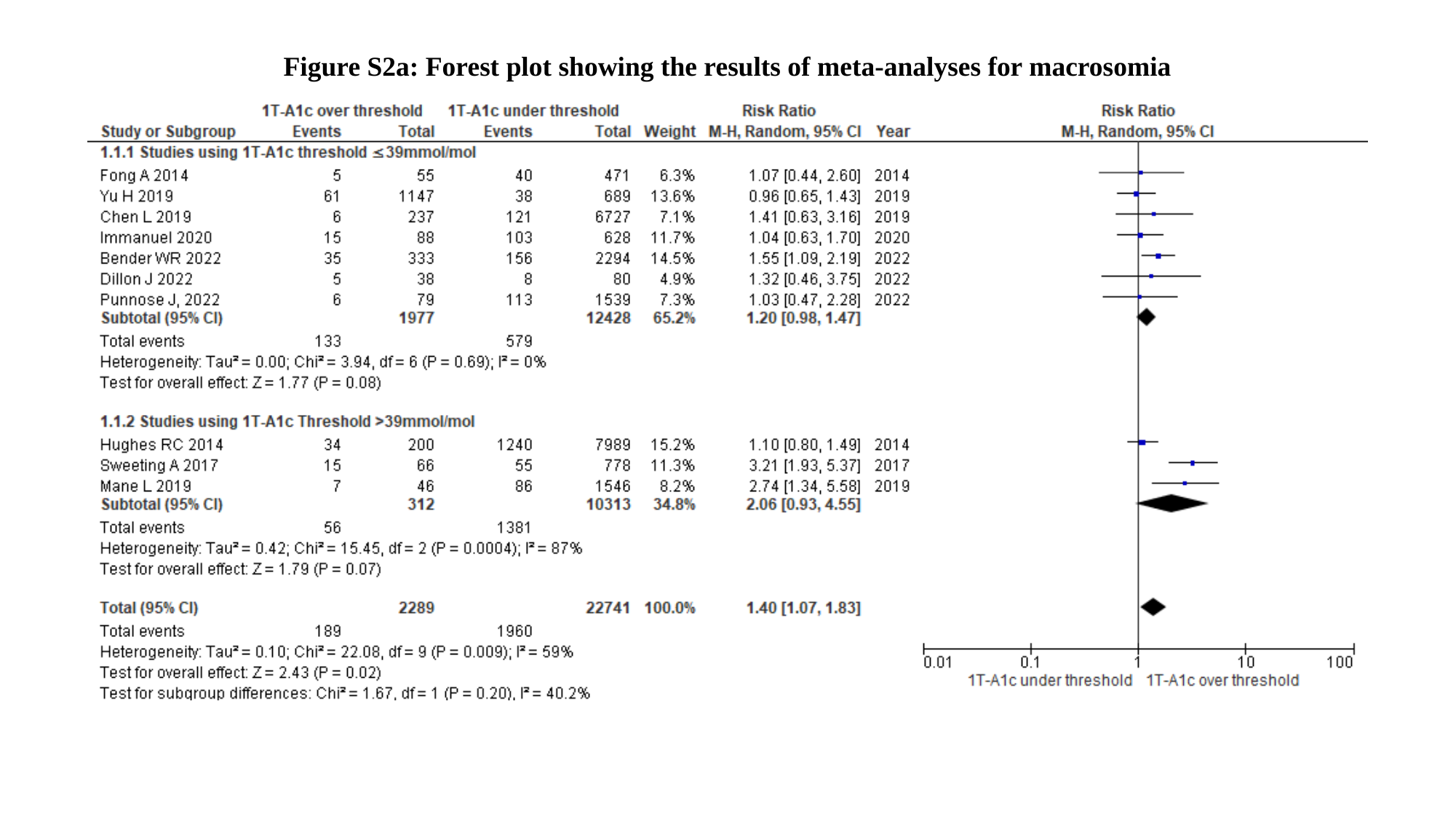

# Figure S2a: Forest plot showing the results of meta-analyses for macrosomia

## Slide 4
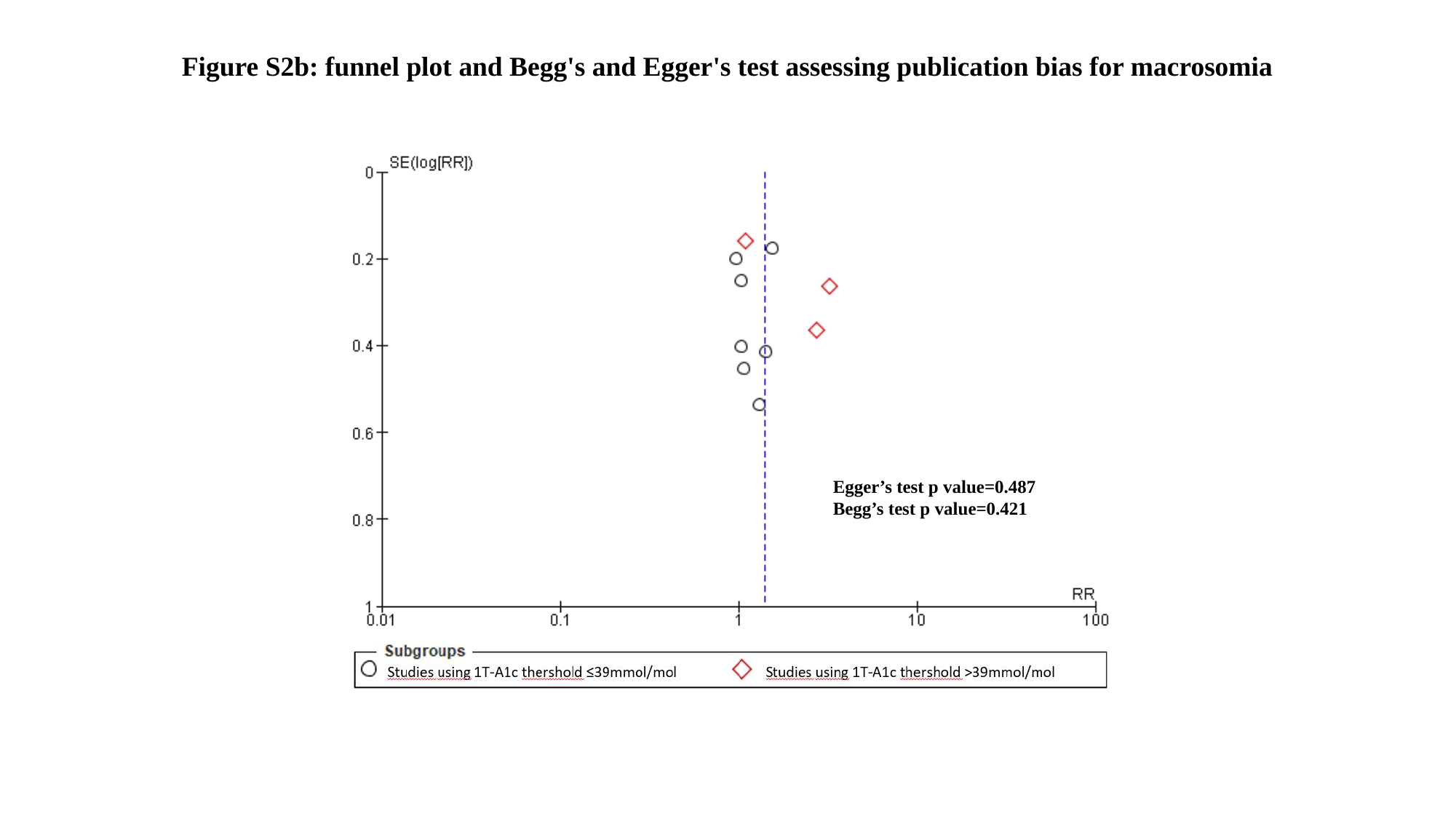

# Figure S2b: funnel plot and Begg's and Egger's test assessing publication bias for macrosomia
Egger’s test p value=0.487
Begg’s test p value=0.421

## Slide 5
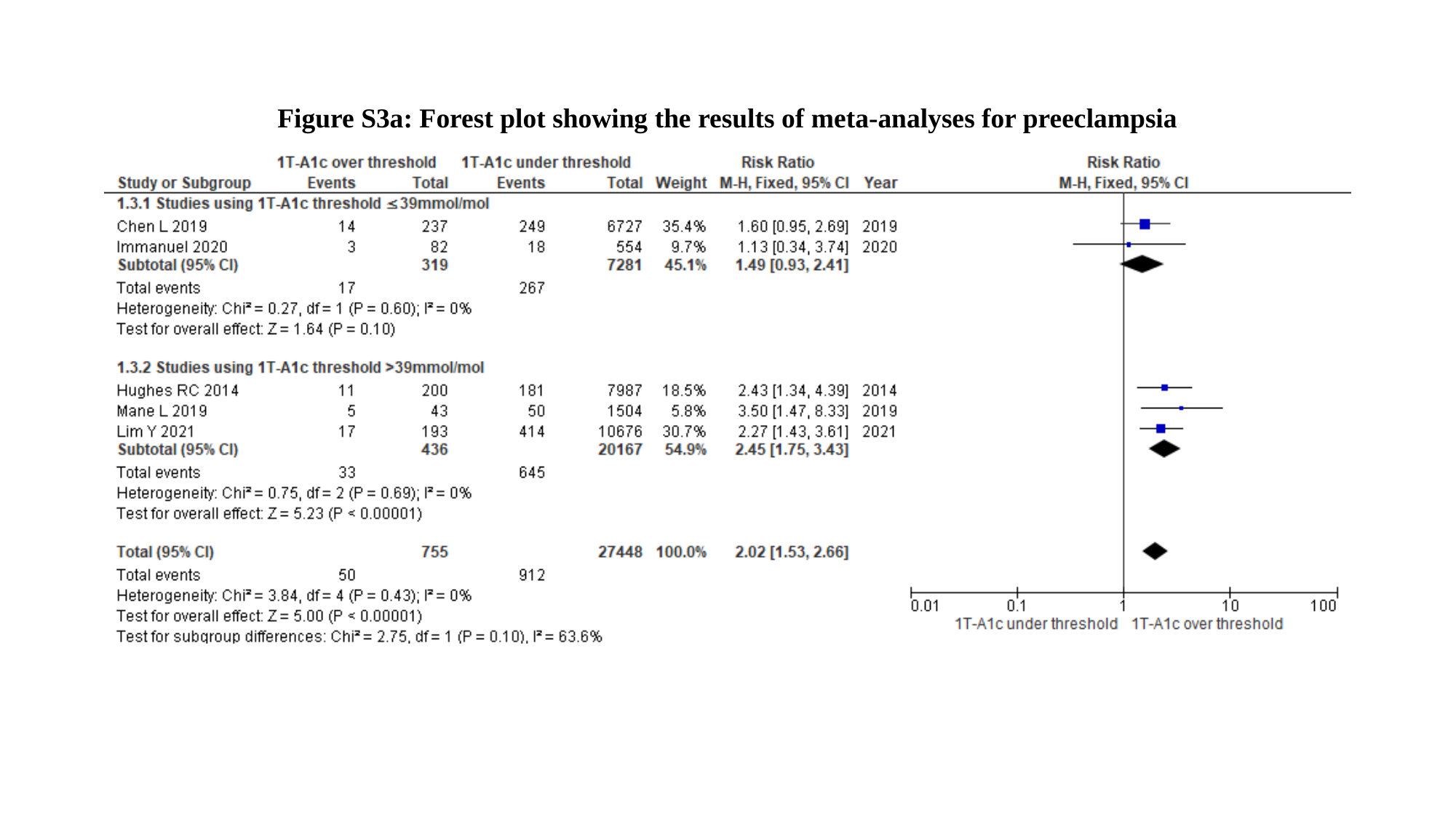

# Figure S3a: Forest plot showing the results of meta-analyses for preeclampsia

## Slide 6
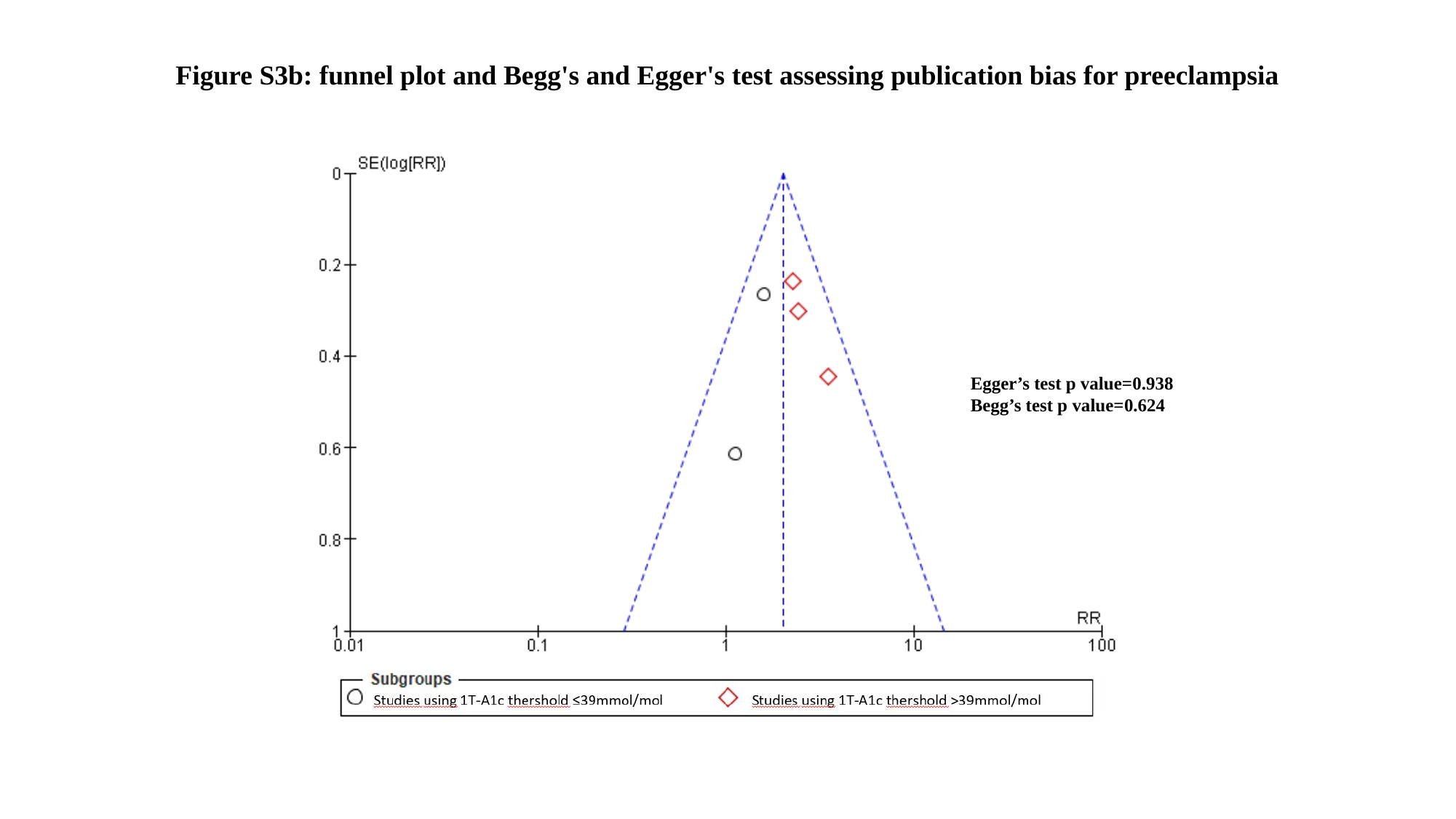

# Figure S3b: funnel plot and Begg's and Egger's test assessing publication bias for preeclampsia
Egger’s test p value=0.938
Begg’s test p value=0.624

## Slide 7
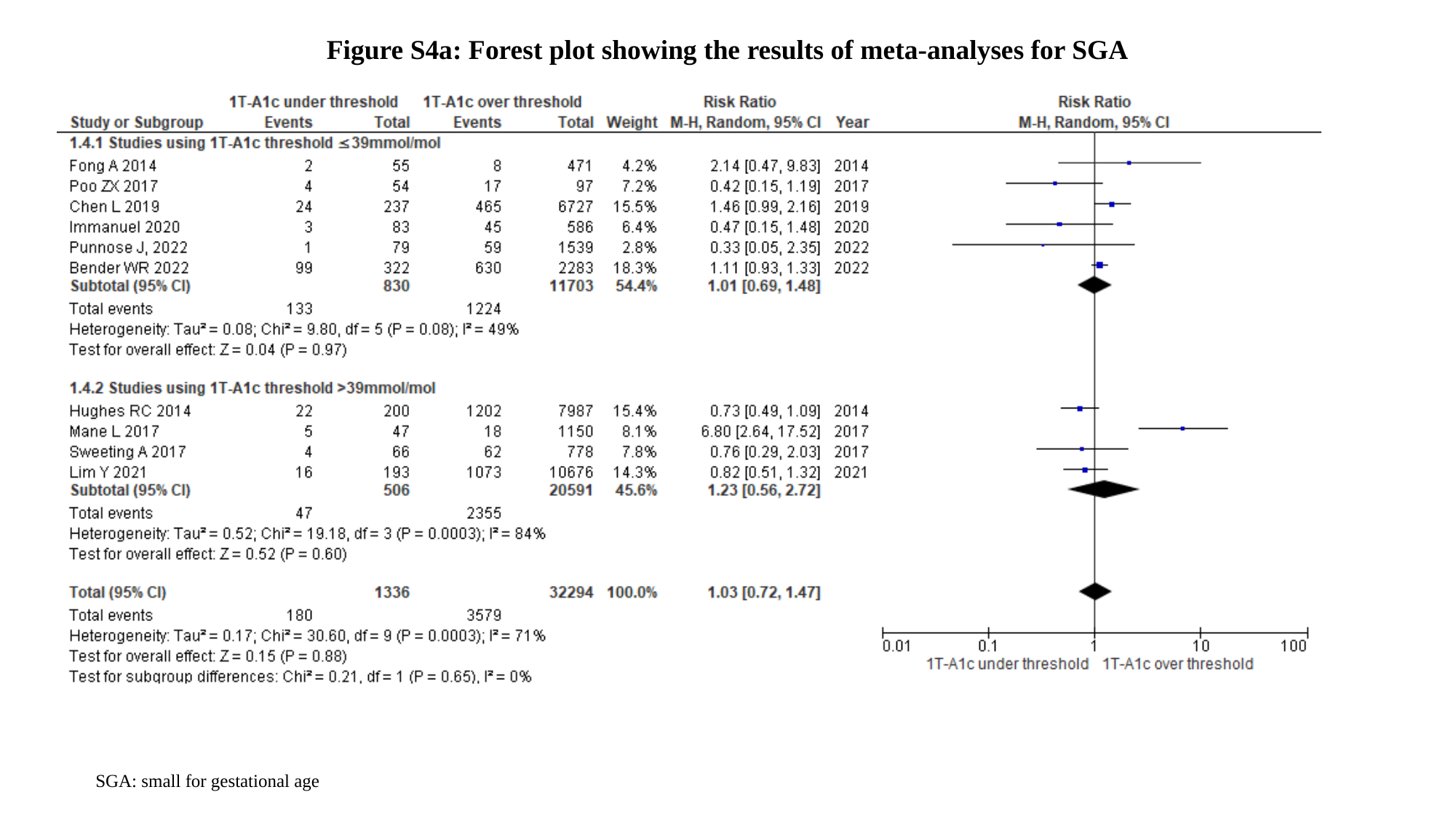

# Figure S4a: Forest plot showing the results of meta-analyses for SGA
SGA: small for gestational age

## Slide 8
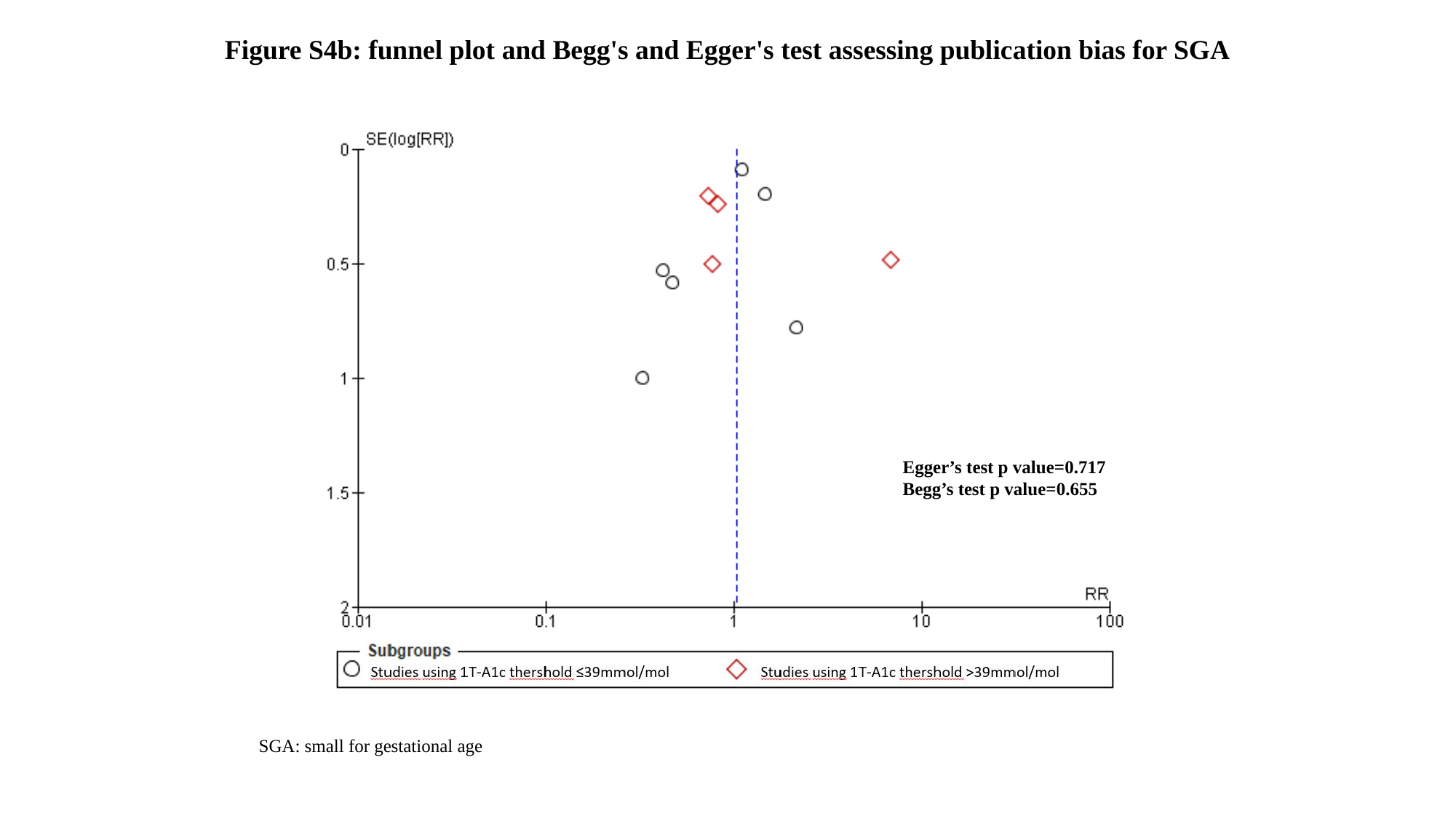

# Figure S4b: funnel plot and Begg's and Egger's test assessing publication bias for SGA
Egger’s test p value=0.717
Begg’s test p value=0.655
Egger’s test p value=0.717
Begg’s test p value=0.655
SGA: small for gestational age

## Slide 9
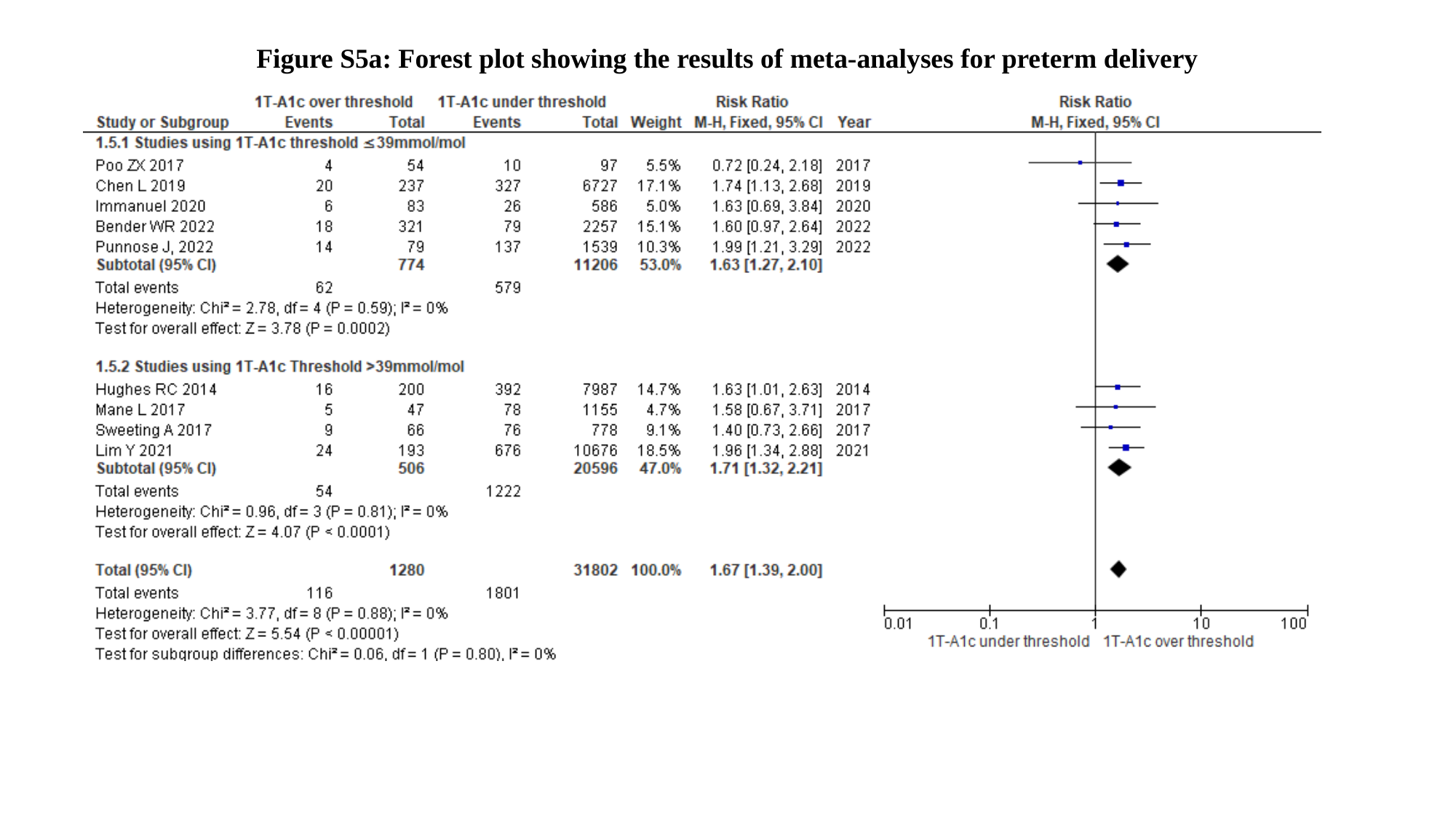

# Figure S5a: Forest plot showing the results of meta-analyses for preterm delivery

## Slide 10
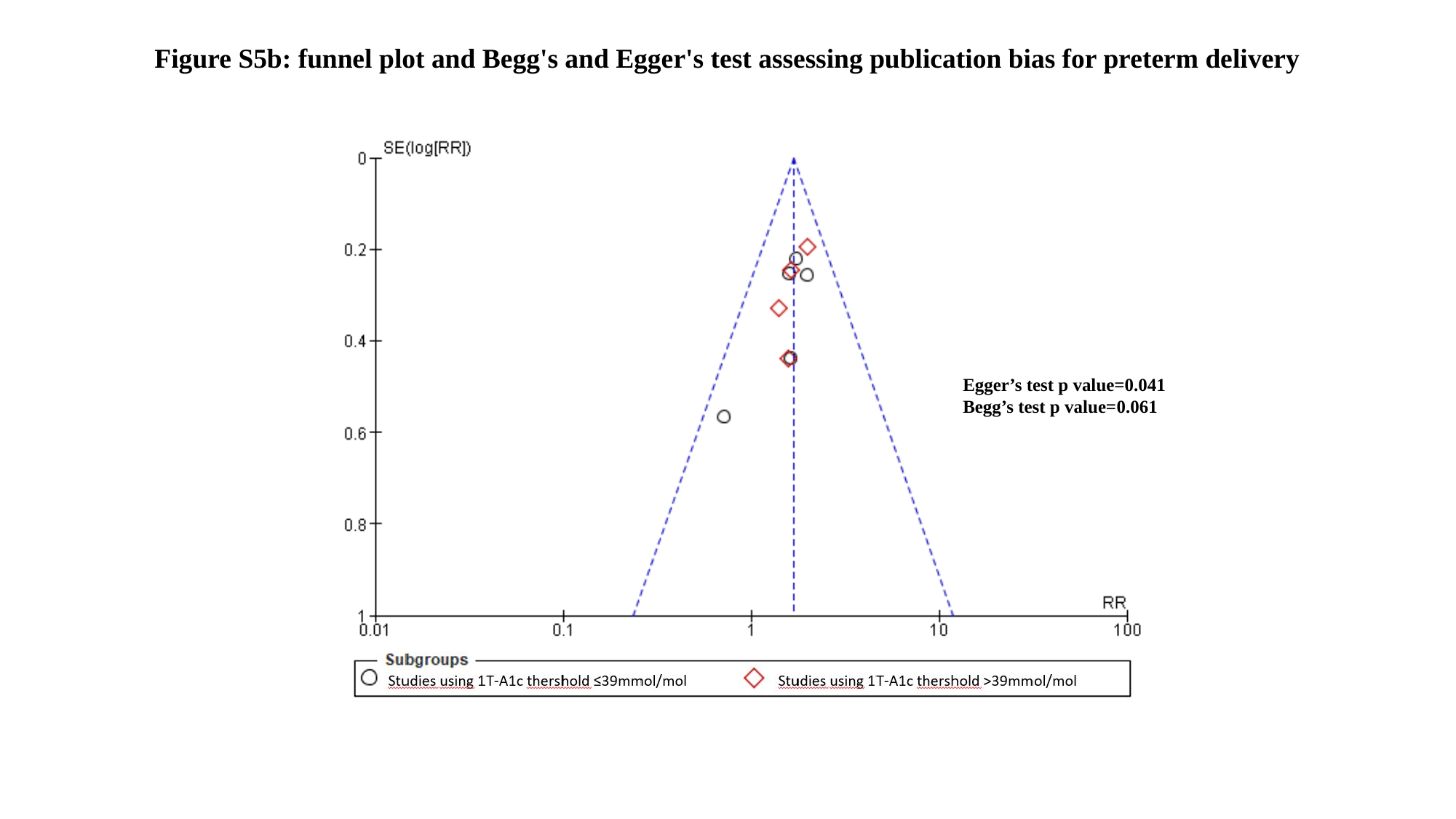

# Figure S5b: funnel plot and Begg's and Egger's test assessing publication bias for preterm delivery
Egger’s test p value=0.041
Begg’s test p value=0.061
Egger’s test p value=0.041
Begg’s test p value=0.061

## Slide 11
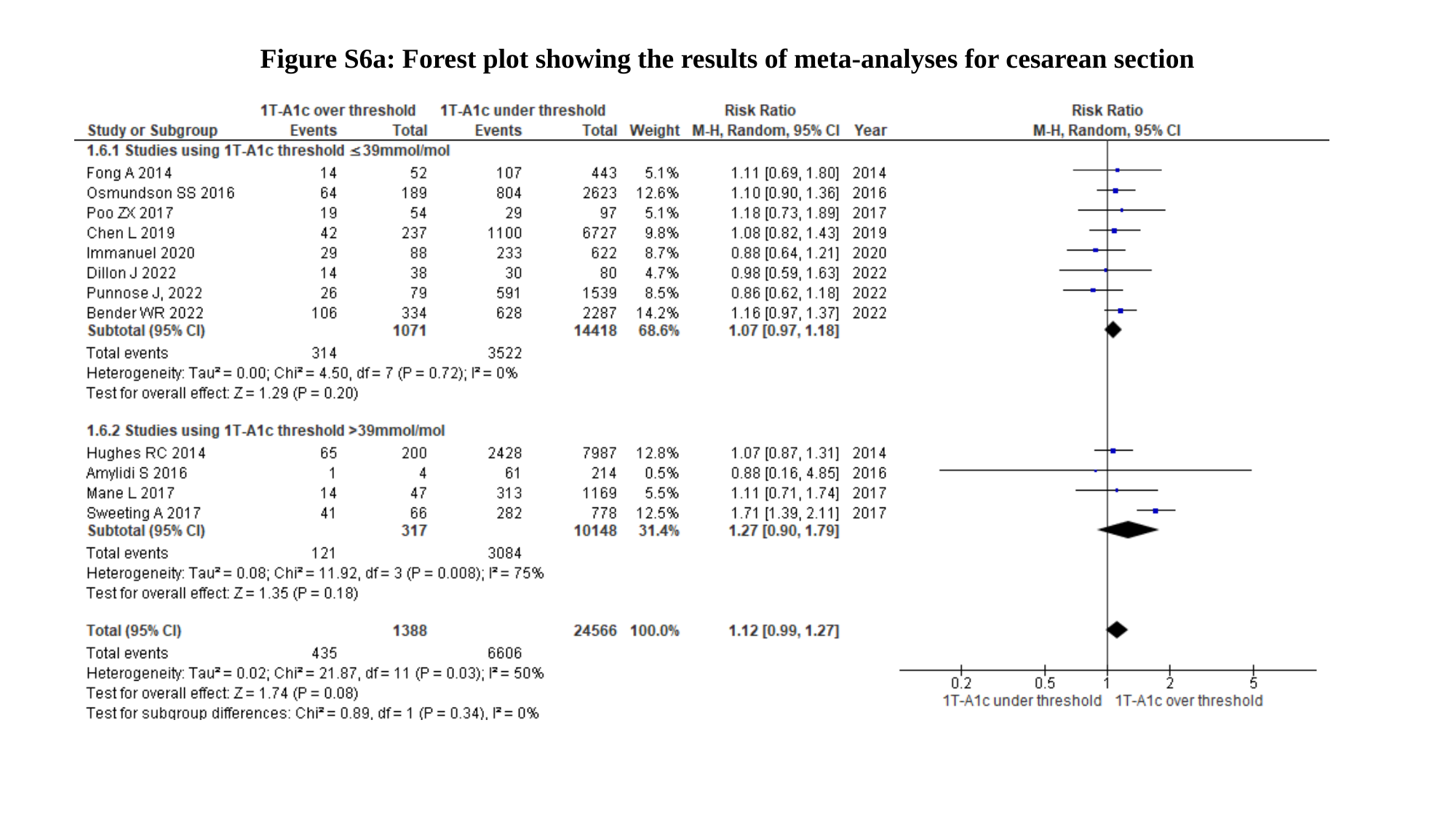

# Figure S6a: Forest plot showing the results of meta-analyses for cesarean section

## Slide 12
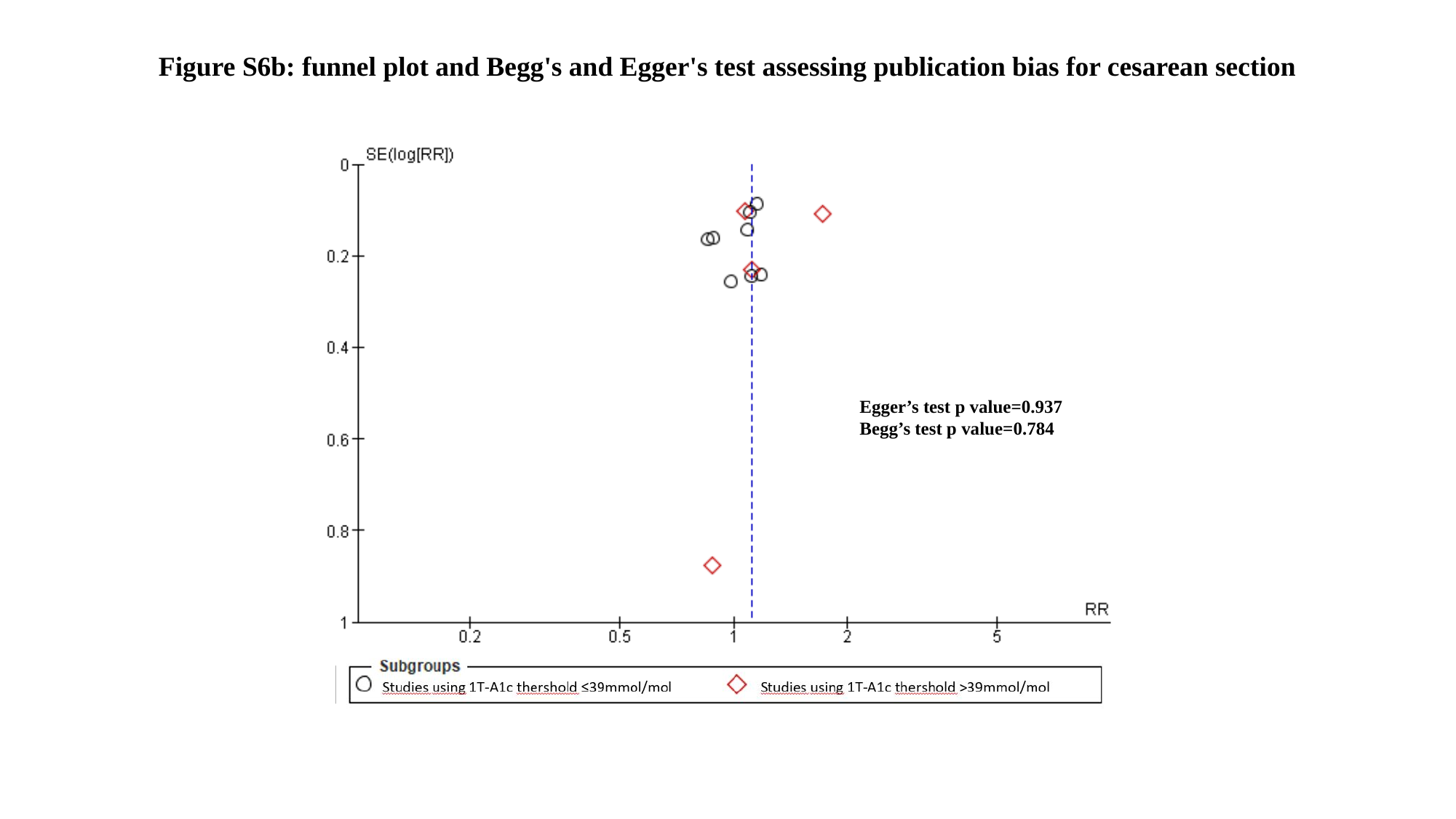

# Figure S6b: funnel plot and Begg's and Egger's test assessing publication bias for cesarean section
Egger’s test p value=0.937
Begg’s test p value=0.784
Egger’s test p value=0.937
Begg’s test p value=0.784

## Slide 13
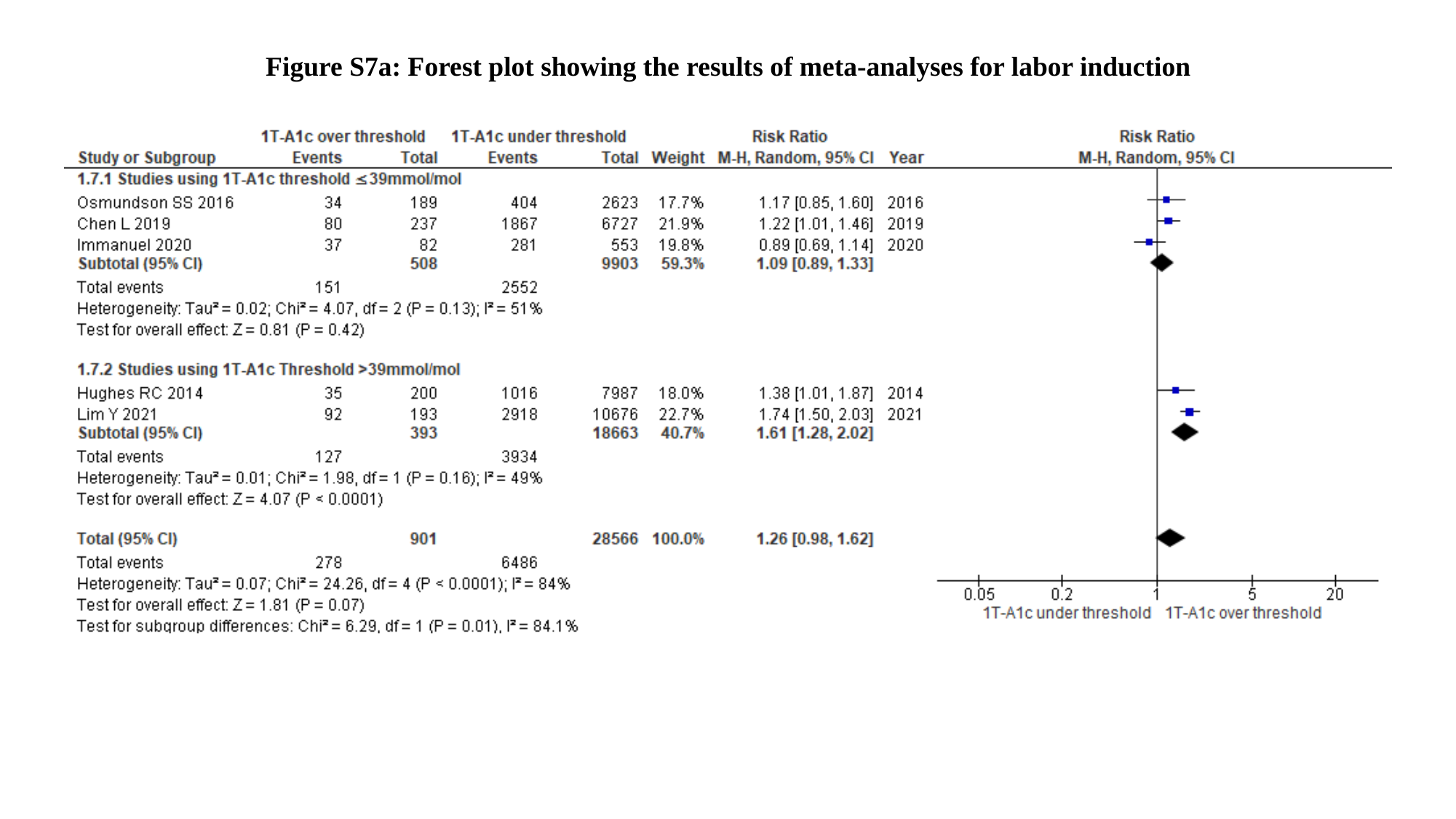

# Figure S7a: Forest plot showing the results of meta-analyses for labor induction

## Slide 14
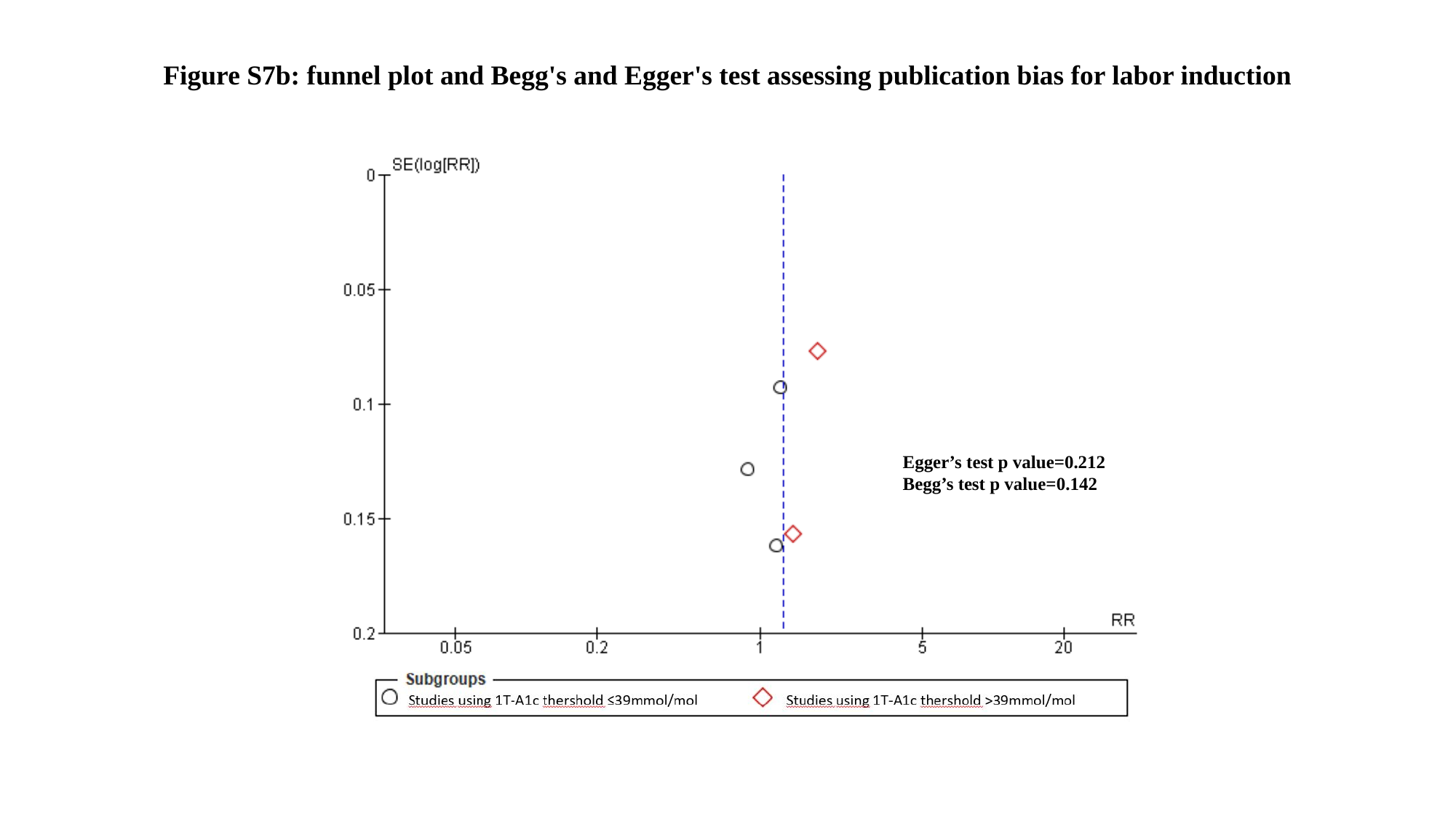

# Figure S7b: funnel plot and Begg's and Egger's test assessing publication bias for labor induction
Egger’s test p value=0.212
Begg’s test p value=0.142
Egger’s test p value=0.212
Begg’s test p value=0.142

## Slide 15
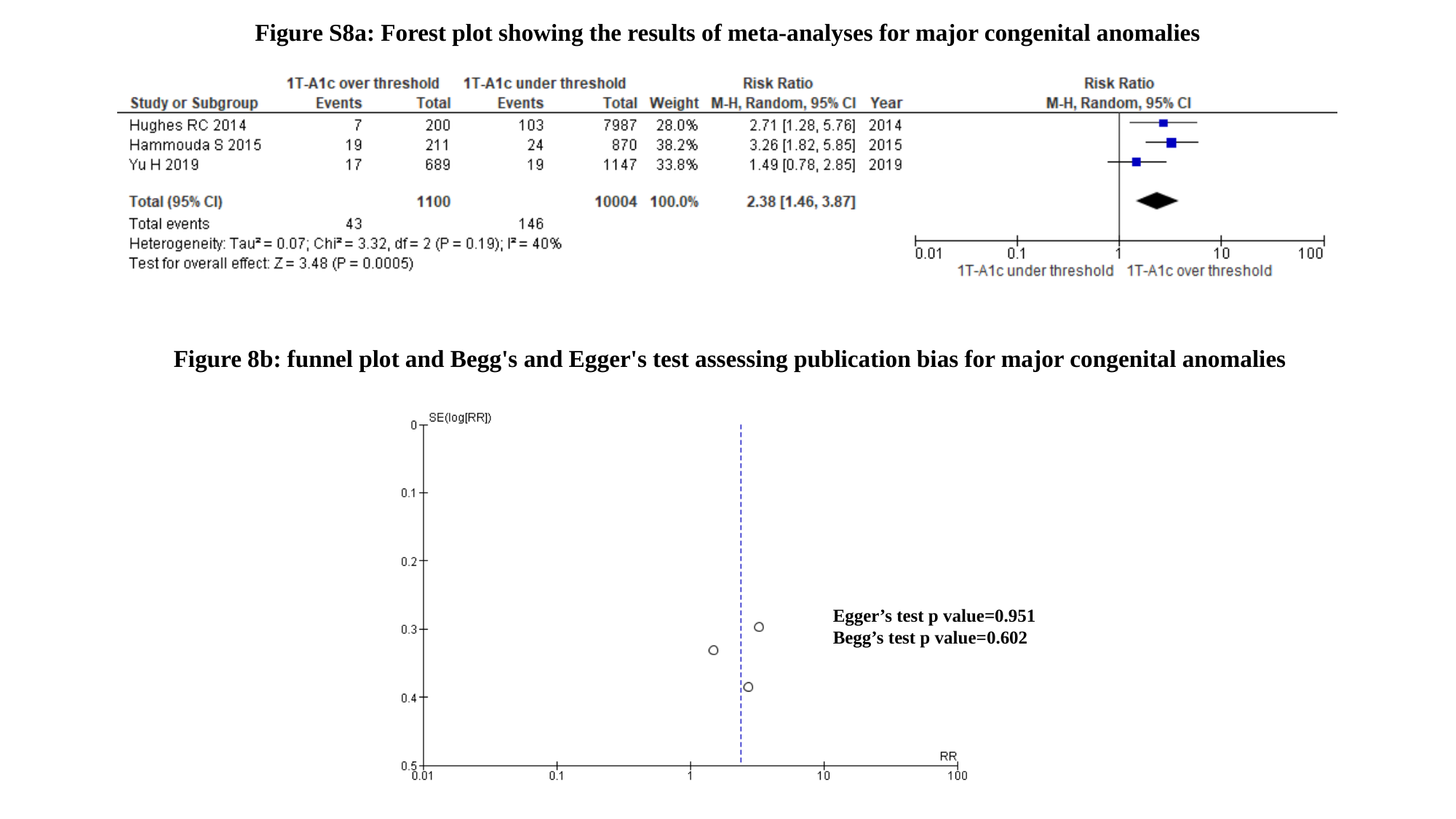

# Figure S8a: Forest plot showing the results of meta-analyses for major congenital anomalies
Figure 8b: funnel plot and Begg's and Egger's test assessing publication bias for major congenital anomalies
Egger’s test p value=0.951
Begg’s test p value=0.602

## Slide 16
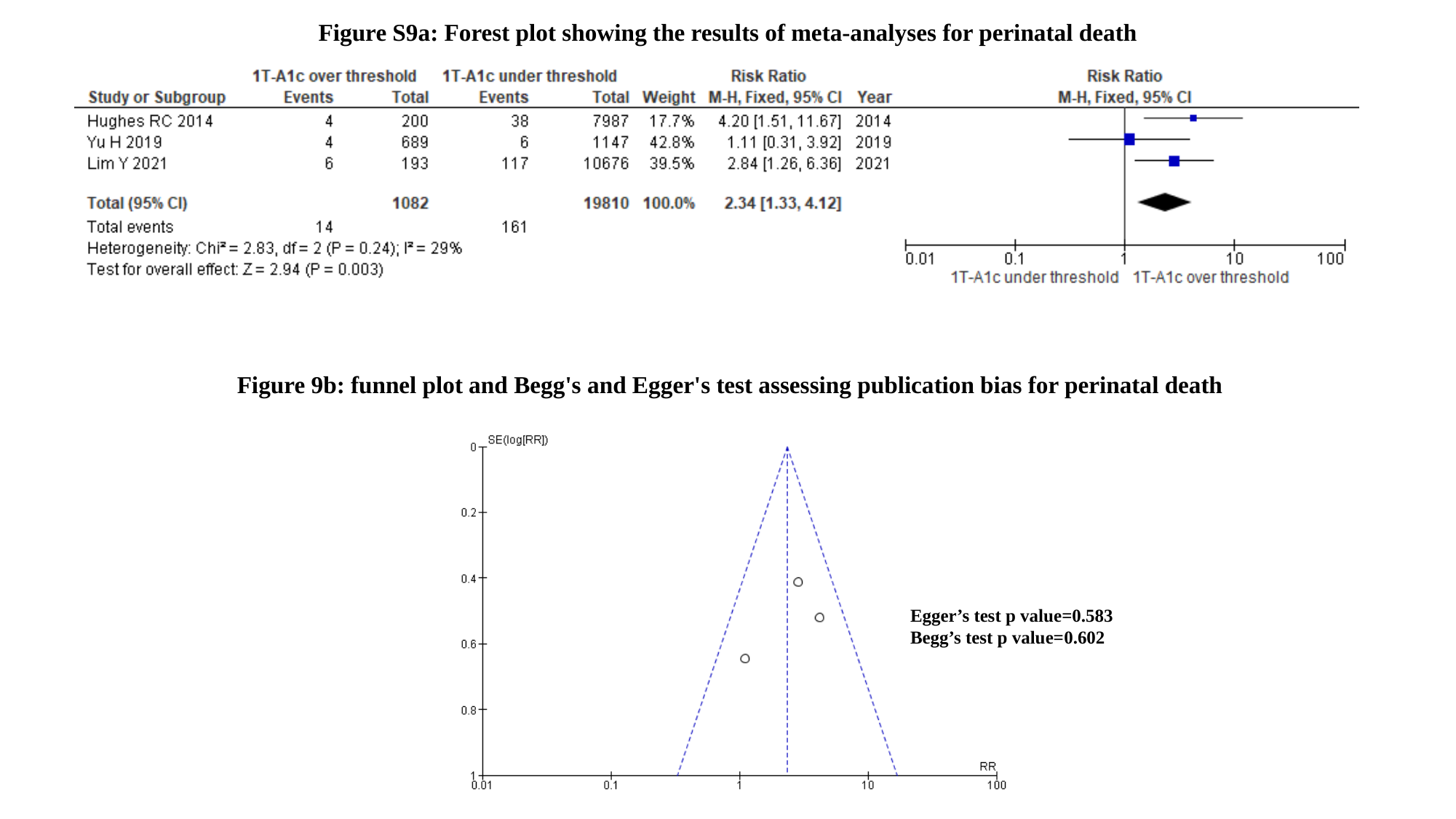

# Figure S9a: Forest plot showing the results of meta-analyses for perinatal death
Figure 9b: funnel plot and Begg's and Egger's test assessing publication bias for perinatal death
Egger’s test p value=0.583
Begg’s test p value=0.602

## Slide 17
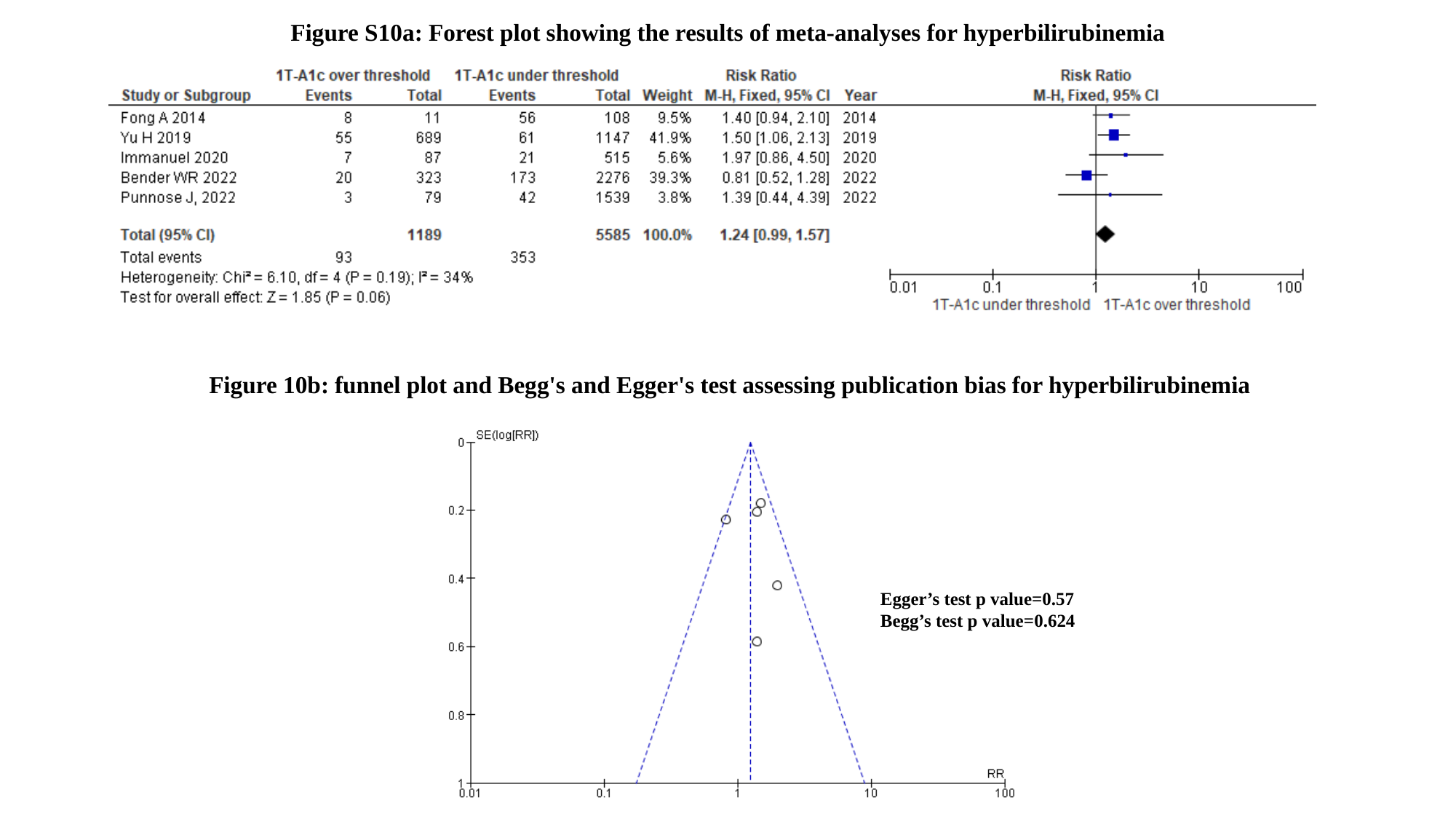

# Figure S10a: Forest plot showing the results of meta-analyses for hyperbilirubinemia
Figure 10b: funnel plot and Begg's and Egger's test assessing publication bias for hyperbilirubinemia
Egger’s test p value=0.57
Begg’s test p value=0.624

## Slide 18
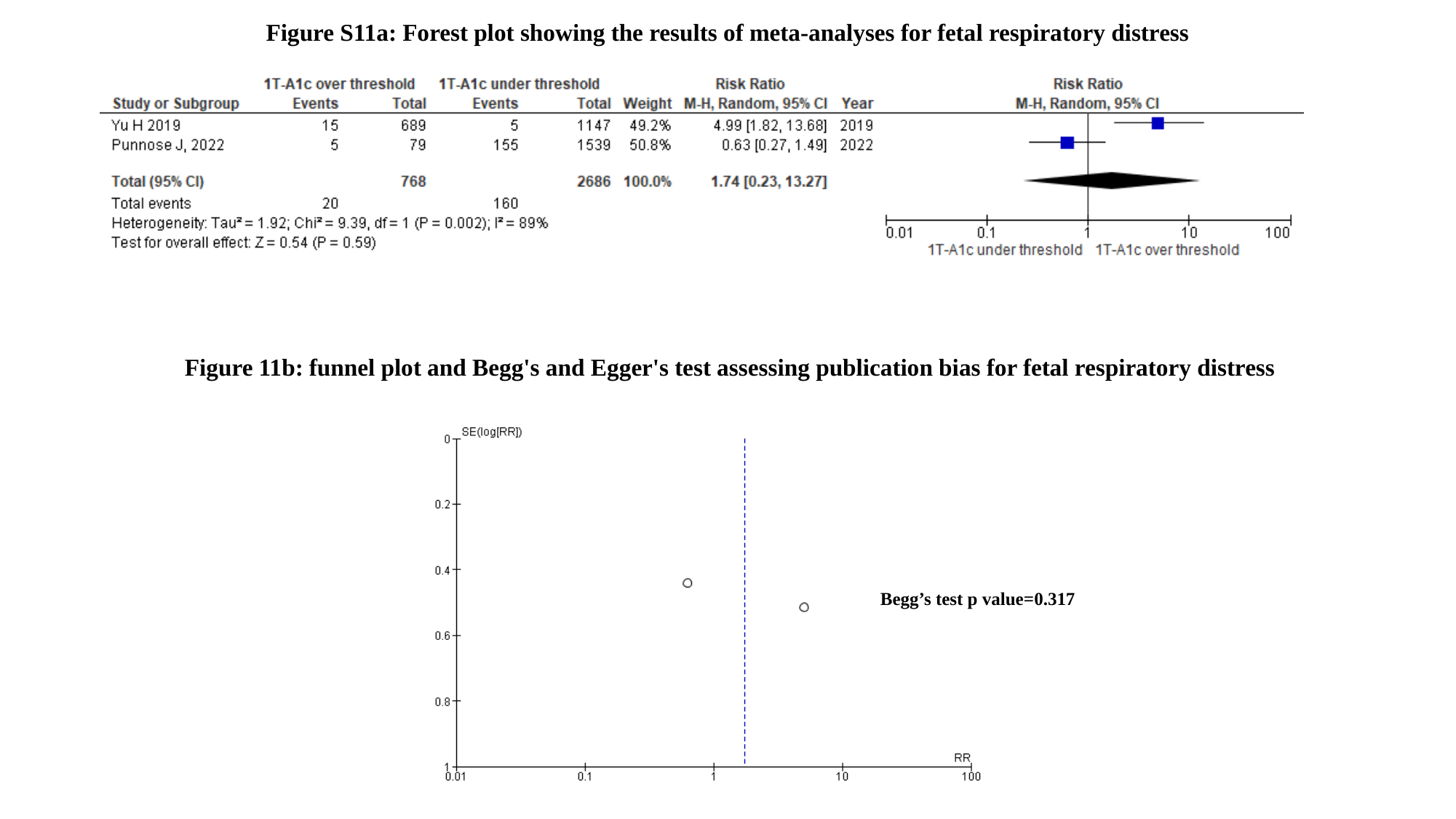

# Figure S11a: Forest plot showing the results of meta-analyses for fetal respiratory distress
Figure 11b: funnel plot and Begg's and Egger's test assessing publication bias for fetal respiratory distress
Begg’s test p value=0.317

## Slide 19
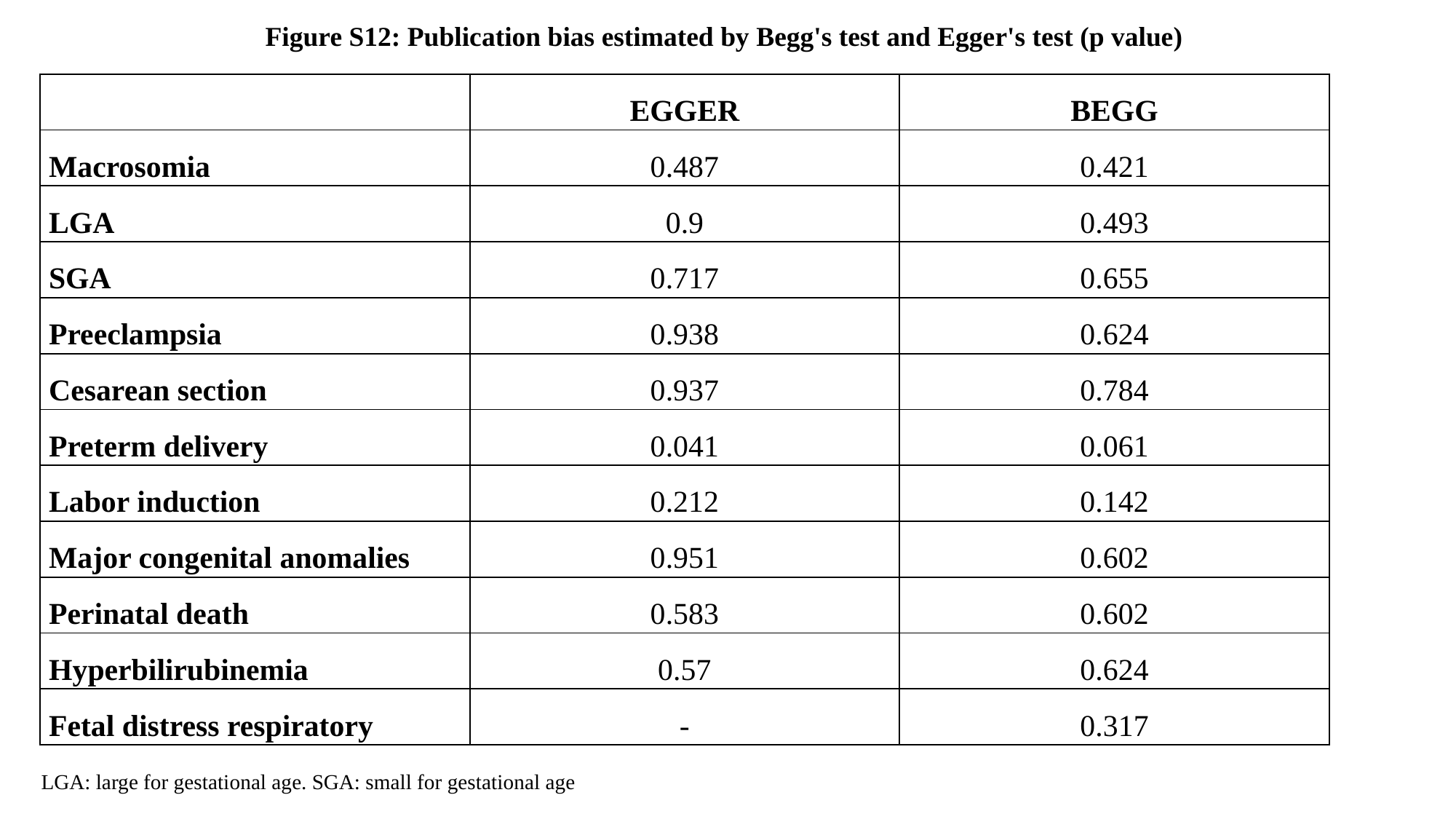

Figure S12: Publication bias estimated by Begg's test and Egger's test (p value)
| | EGGER | BEGG |
| --- | --- | --- |
| Macrosomia | 0.487 | 0.421 |
| LGA | 0.9 | 0.493 |
| SGA | 0.717 | 0.655 |
| Preeclampsia | 0.938 | 0.624 |
| Cesarean section | 0.937 | 0.784 |
| Preterm delivery | 0.041 | 0.061 |
| Labor induction | 0.212 | 0.142 |
| Major congenital anomalies | 0.951 | 0.602 |
| Perinatal death | 0.583 | 0.602 |
| Hyperbilirubinemia | 0.57 | 0.624 |
| Fetal distress respiratory | - | 0.317 |
LGA: large for gestational age. SGA: small for gestational age
