# Supplementary material for: Early HbA1c Levels as a Predictor of Adverse Obstetric Outcomes: A Systematic Review and Meta-Analysis
Source: J Clin Med. 2024 Mar 17;13(6):1732. doi: 10.3390/jcm13061732 (PMC10970986; doi:10.3390/jcm13061732)
Supplement: Supplementary file 1 [file jcm-13-01732-s001.zip › Table S1 (search strategy).pdf]

Supplementary Table S1: Search strategy

**MEDLINE**<br>Ovid MEDLINE(R) and Epub Ahead of Print, In-Process, In-Data-Review & Other Non-Indexed Citations, Daily and Versions <1946 to October 20, 2022>	1 Glycated Hemoglobin A/<br>2 Pregnancy Trimester, First/<br>3 Pregnancy/<br>4 Fetal Macrosomia/<br>5 Pre-Eclampsia/<br>6 Premature Birth/<br>7 Pregnancy Outcome/<br>8 Infant, Small for Gestational Age/<br>9 Gestational Age/<br>10 Cesarean Section/<br>11 Labor, Induced/<br>12 Congenital Abnormalities/<br>13 Hyperbilirubinemia, Neonatal/<br>14 Perinatal Death/<br>15 2 or 3<br>16 4 or 5 or 6 or 7 or 8 or 9 or 10 or 11 or 12 or 13 or 14<br>17 1 and 15 and 16<br>18 limit 17 to (humans and (English or Portuguese or Spanish))
**EMBASE**<br><1974 to 2022 October 20>	('glycosylated hemoglobin'/exp OR 'glycosylated hemoglobin') AND ('first trimester pregnancy'/exp OR 'first trimester pregnancy') AND ('macrosomia'/exp OR 'macrosomia' OR 'preeclampsia'/exp OR 'preeclampsia' OR 'prematurity'/exp OR 'prematurity' OR 'pregnancy complication'/exp OR 'pregnancy complication' OR 'small for date infant'/exp OR 'small for date infant' OR 'gestational age'/exp OR 'gestational age' OR 'cesarean section'/exp OR 'cesarean section' OR 'labor induction'/exp OR 'labor induction' OR 'congenital disorder'/exp OR 'congenital disorder' OR 'neonatal hyperbilirubinemia'/exp OR 'neonatal hyperbilirubinemia' OR 'perinatal death'/exp OR 'perinatal death')
