# Supplementary material for: Early HbA1c Levels as a Predictor of Adverse Obstetric Outcomes: A Systematic Review and Meta-Analysis
Source: J Clin Med. 2024 Mar 17;13(6):1732. doi: 10.3390/jcm13061732 (PMC10970986; doi:10.3390/jcm13061732)
Supplement: Supplementary file 1 [file jcm-13-01732-s001.zip › Table S2 (risk of bias - robins).pdf]

Supplementary Table S2: Risk of bias for the included observational studies using the ROBINS-I tool

	Confounding	Selection of participants	Classification of interventions	Deviations from the intended intervention	Missing data	Measurement of outcomes	Selection of reported result	Overall risk of bias
**Hughes RC, 2014**[15]	Low	Low	No information	No information	Low	Low	Low	Low
**Fong A, 2014**[22]	Low	Low	No information	No information	Moderate	Low	Low	Moderate
**Hammouda S, 2015**[20]	Low	Moderate	No information	No information	Low	Low	Low	Moderate
**Amylidi S, 2016**[29]	Low	Moderate	No information	No information	Moderate	Low	Low	Moderate
**Osmundson SS, 2016**[25]	Low	Low	No information	No information	Low	Low	Low	Low
**Sweeting A, 2017**[17]	Low	Moderate	No information	No information	Moderate	Low	Low	Moderate
**Mane L, 2017**[16]	Low	Low	No information	No information	Low	Low	Low	Low
**Poo ZX, 2018**[26]	Low	Low	No information	No information	Moderate	Low	Low	Moderate
**Yu H, 2019**[28]	Low	Moderate	No information	No information	Low	Low	Low	Moderate
**Chen L, 2019**[19]	Low	Moderate	No information	No information	Low	Low	Low	Moderate
**Mañé L, 2019**[36]	Low	Low	No information	No information	Low	Low	Low	Low
**Immanuel JJ, 2020**[21]	Low	Moderate	No information	No information	Low	Low	Low	Moderate
**Lim Y, 2021**[18]	Low	Low	No information	No information	Moderate	Low	Low	Moderate

**Jamieson EL, 2021**[27]	Moderate	Low	No information	No information	Low	Low	Low	Moderate
**Punnose J, 2022**[30]	Low	Low	No information	No information	Low	Low	Low	Low
**Dillon J, 2022**[23]	Low	Moderate	No information	No information	Moderate	Low	Low	Moderate
**Bender WR, 2022**[24]	Low	Low	No information	No information	Low	Low	Low	Low
